# Two-dimensional digital photography for child body posture evaluation: standardized technique, reliable parameters and normative data for age 7-10 years

**DOI:** 10.1186/s13013-017-0146-7

**Published:** 2017-12-19

**Authors:** L. Stolinski, M. Kozinoga, D. Czaprowski, M. Tyrakowski, P. Cerny, N. Suzuki, T. Kotwicki

**Affiliations:** 1 Department of Spine Disorders and Pediatric Orthopedics, University of Medical Sciences, 28 Czerwca 1956r. no. 135/147, 61-545 Poznan, Poland; 2 Rehasport Clinic, Poznan, Poland; 3 Rehasport Clinic Licensed Rehabilitation Center, Skierniewice, Poland; 4 Department of Physiotherapy, Józef Rusiecki University College, Olsztyn, Poland; 5 Center of Body Posture, Olsztyn, Poland; 6 Department of Orthopaedics, Pediatric Orthopaedics and Traumatology, The Centre of Postgraduate Medical Education in Warsaw, Otwock, Poland; 7 Faculty of Health Studies, University of West Bohemia, Pilsen, Czech Republic; 8 Faculty of Physical Education and Sport, Charles University, Prague, Czech Republic; 9 ORTOTIKA, s. r. o, Faculty at Motol University Hospital, Prague, Czech Republic; 10 Scoliosis Center, Medical Scanning Tokyo, Tokyo, Japan

**Keywords:** Standardization, Digital photography, Photogrammetry, Percentile charts, Normative data, Primary school children

## Abstract

**Background:**

Digital photogrammetry provides measurements of body angles or distances which allow for quantitative posture assessment with or without the use of external markers. It is becoming an increasingly popular tool for the assessment of the musculoskeletal system. The aim of this paper is to present a structured method for the analysis of posture and its changes using a standardized digital photography technique.

**Material and methods:**

The purpose of the study was twofold. The first one comprised 91 children (44 girls and 47 boys) aged 7–10 (8.2 ± 1.0), i.e., students of primary school, and its aim was to develop the photographic method, choose the quantitative parameters, and determine the intraobserver reliability (repeatability) along with the interobserver reliability (reproducibility) measurements in sagittal plane using digital photography, as well as to compare the Rippstein plurimeter and digital photography measurements. The second one involved 7782 children (3804 girls, 3978 boys) aged 7–10 (8.4 ± 0.5), who underwent digital photography postural screening. The methods consisted in measuring and calculating selected parameters, establishing the normal ranges of photographic parameters, presenting percentile charts, as well as noticing common pitfalls and possible sources of errors in digital photography.

**Results:**

A standardized procedure for the photographic evaluation of child body posture was presented. The photographic measurements revealed very good intra- and inter-rater reliability regarding the five sagittal parameters and good reliability performed against Rippstein plurimeter measurements. The parameters displayed insignificant variability over time. Normative data were calculated based on photographic assessment, while the percentile charts were provided to serve as reference values. The technical errors observed during photogrammetry are carefully discussed in this article.

**Conclusions:**

Technical developments are allowed for the regular use of digital photogrammetry in body posture assessment. Specific child positioning (described above) enables us to avoid incidentally modified posture. Image registration is simple, quick, harmless, and cost-effective. The semi-automatic image analysis, together with the normal values and percentile charts, makes the technique reliable in terms of child’s posture documentation and corrective therapy effects’ monitoring.

**Electronic supplementary material:**

The online version of this article (10.1186/s13013-017-0146-7) contains supplementary material, which is available to authorized users.

## Background

### Human body posture

Body posture is defined as the alignment of body segments which is considered as an important health indicator [1]. Human body posture is also described as a motor habit accompanying daily activities [2]. Normal human posture is the characteristic of the vertical position which relies on spinal alignment and its position over the patient’s head and pelvis [3, 4]. Human body posture undergoes large variability, which depends on age, sex, body growth, environmental factors, and psychophysical status of an individual [5–7]. The accurate description of human body posture represents a topic of interest for the scientists aiming to measure and to document the posture. For the clinicians, posture evaluation plays a role in the global health assessment. On the one hand, faulty posture may result from various disorders, while the posture itself may be even patognomic for certain diseases (ex. spondylolisthesis). On the other hand, incorrect body posture can have negative impact on the overall health, leading to pain or functional disorder, which means that it can affect the quality of life both in childhood and adulthood [8].

The quality of body posture results from individual settings of respective body parts, especially the spine [9] and pelvis [10] alignment in the sagittal plane. The gravity line is defined as the vertical line passing through the center of gravity in the entire body. For a standing subject, the reference posture is described by the relations between the gravity line and body segments [11]. Balanced arrangement of body parts provides the basis for the center of mass. Such arrangement of body parts enables the maintenance of horizontal gaze as well as effective muscle contraction and stretching without unnecessary loss of energy [12]. Diagnostic tools for measuring the sagittal spine curvatures and the pelvis alignment can be used to describe a correct posture while standing [13].

The multitude of methods and diagnostic tools makes it difficult to standardize the assessment of body posture. In addition, there is a lack of a clear range between the traditional and faulty posture—in particular, the number of quantitative posture parameters. Thus, the data on the prevalence of faulty posture is very divergent and based on different diagnostic criteria [14].

The content of the paper fulfills the following objectives: (1) to standardize digital photography technique for posture assessment; (2) to determine the intra-observer reproducibility and the inter-observer reliability of photographic sagittal parameters: sacral slope (SS), lumbar lordosis (LL), thoracic kyphosis (TK), chest inclination (CI), and head protraction (HP); (3) to check the validity of photographic measurements against the Rippstein plurimeter measurements; (4) to analyze the variability of five sagittal photographic angles: SS, LL, TK, CI, HP, and two coronal parameters: Anterior Trunk Symmetry Index (ATSI) and Posterior Trunk Symmetry Index (POTSI) over time (1 week); (5) to present the normative values of sagittal photographic parameters based on photographic assessment of 7782 children aged 7–10; and (6) to discuss common pitfalls and sources of errors in digital photography used in posture evaluation.

## Methods

### Standardization of posture assessment with digital photography

The use of reliable tools and methods for clinical measurements is the first step towards evidence-based medicine [15] as the foundation of effective and safe clinical practice. Just like any tool, the photographic technique for posture evaluation should be checked and validated before use. Standardization required to assess body posture was performed as part of this study.

#### Preparing a patient to photogrammetry

##### Marking anatomical body landmarks

**Fig. 1:**
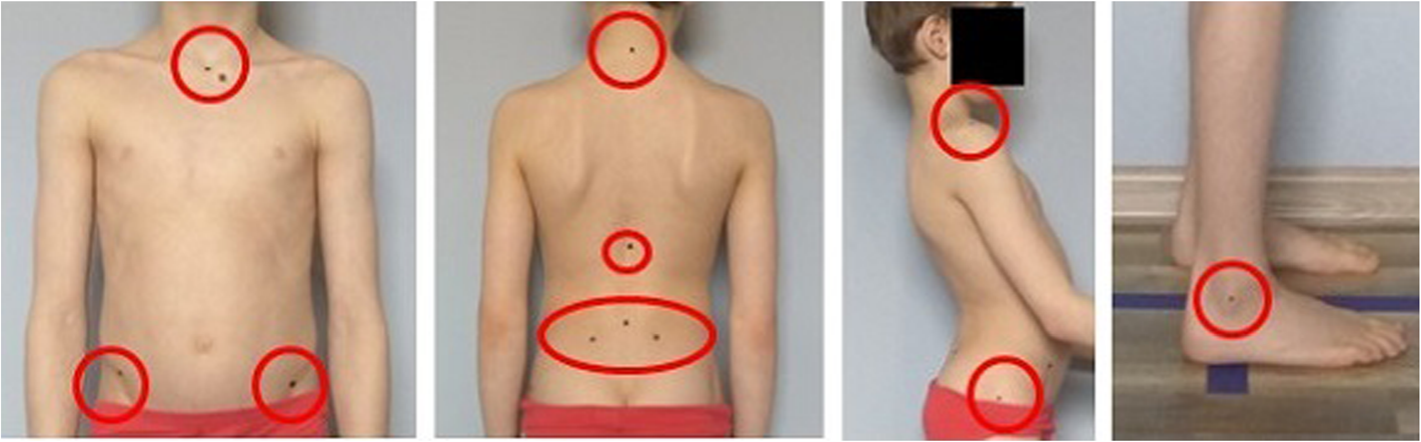
Anatomical points marked on the body

In the procedure below, body posture is assessed without the use of external markers attached to the skin. Dots corresponding to the anatomical body landmarks are drawn on the skin with the use of a non-toxic color pencil. The following body landmarks are marked (Fig. 1):

- The center of the sternal notch
- Anterior superior iliac spine (ASIS)—right and left
- Posterior superior iliac spine (PSIS)—right and left
- Spinous process of C7
- The point between T12 and L1 spinous process
- The point between L5 and S1 spinous process
- The center of acromion—right and left
- The center of greater trochanter—right and left
- The center of external malleolus of the ankle joint

##### Positioning the patient

**Fig. 2:**
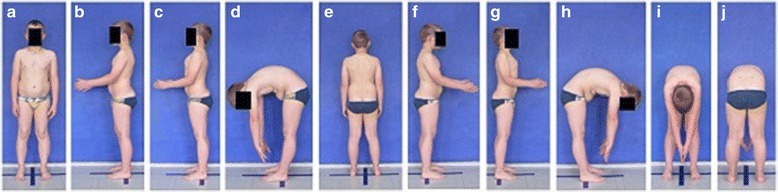
**a**–**j** Standardized positions for posture photogrammetry

**Positioning children during body posture evaluation** Standardized procedure for photographic body posture evaluation includes the photos presented in Fig. 2: spontaneous standing frontal posture (2a), sagittal profiles including photos of the left side (2b), left side actively corrected (2c), left side in forward bending (2d), spontaneous standing posture of the back (2e), right side (2f), right side actively corrected (2g), right side in forward bending (2h), as well as front (2i) and back forward bending (2j).

**Positioning children during scoliosis rib and lumbar prominence evaluation** In order to document the angle of trunk rotation at different trunk levels, one can take a sequence of photos (5–15) made during forward bending of a child (Fig. 3).

**Fig. 3:**
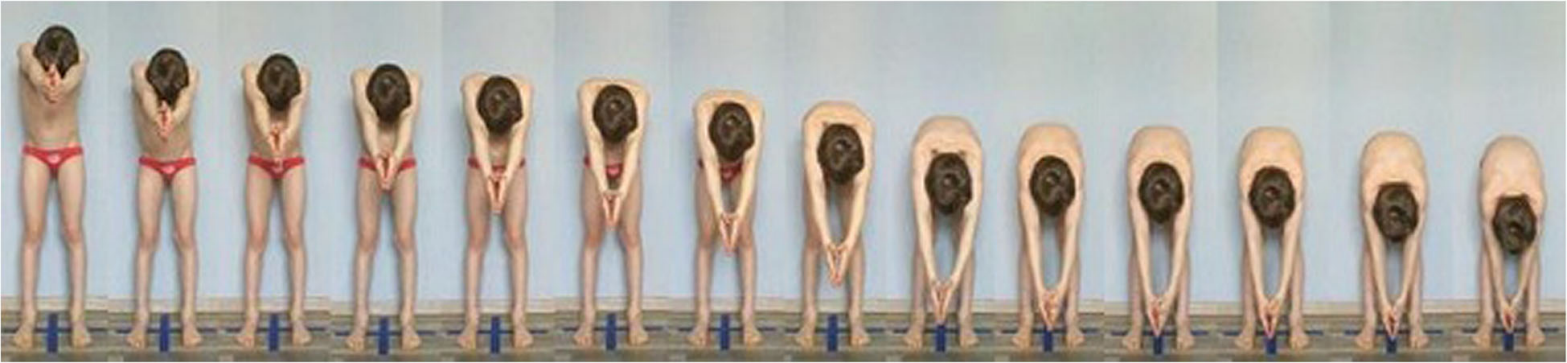
Photographic documentation of trunk rotation/trunk inclination deformity revealed during Adams’ forward bending test (left to right—progressive forward bending)

**Fig. 4:**
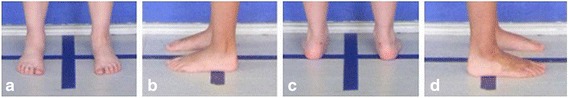
Feet positioning for posture photographic evaluation: **a** front view, **b** lateral left view, **c** back view, and **d** lateral right view

**Lower limb positioning in photographic examination** The undressed child (wearing the underwear and a narrow bra for girls) is barefoot with its knees extended and the feet hip-width apart. The feet are placed on longitudinal and crosswise lines marked on the ground so that their lateral malleoli are situated over the center of the crosswise line and the feet stay parallel to the longitudinal line (Fig. 4). Most of the upper part of the intergluteal cleft should be uncovered.

**Fig. 5:**
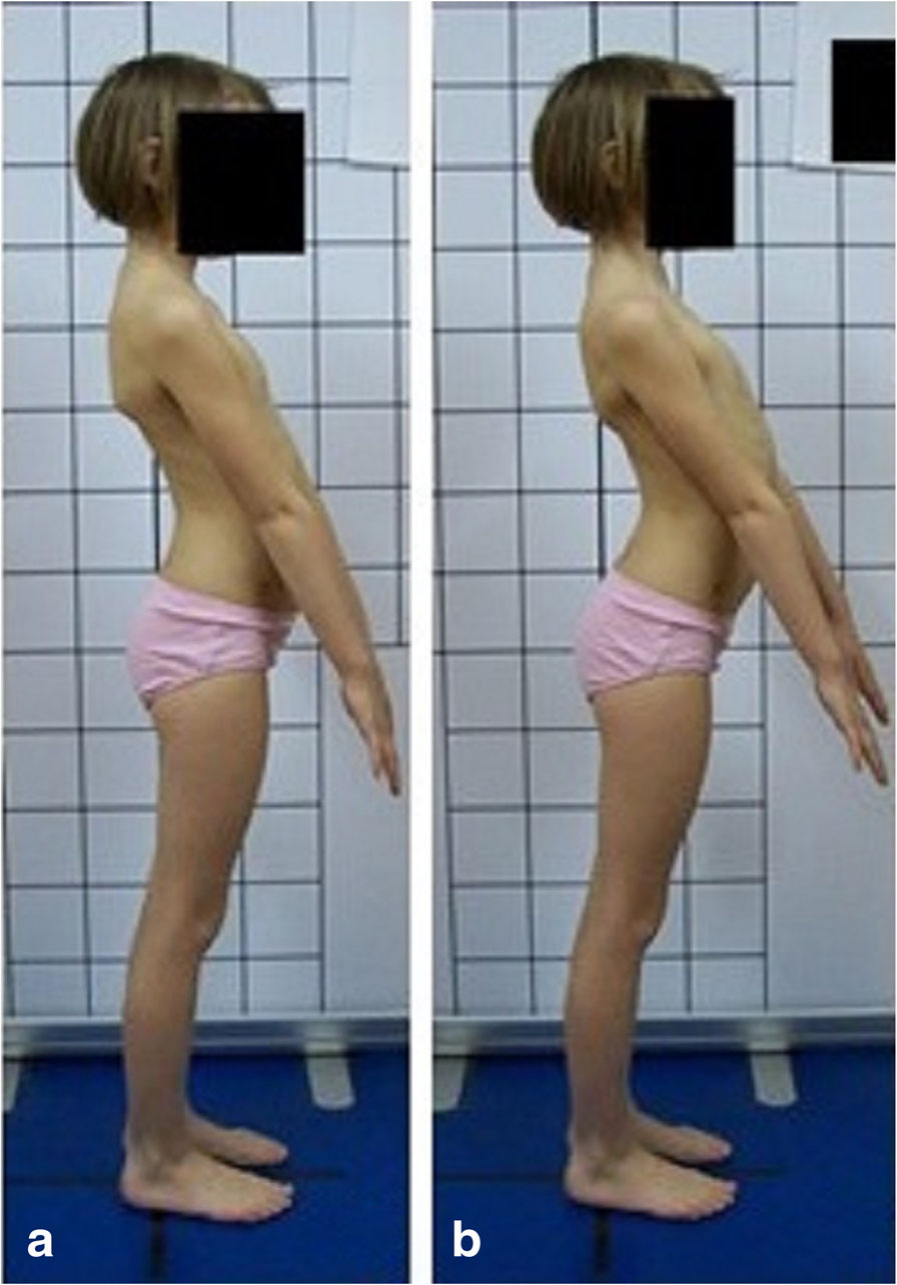
Child’s posture taken for sagittal plane assessment: **a** spontaneous standing posture, **b** “actively corrected posture” in this child reveals backward trunk hyperextension which should be avoided; such image points out the importance of children education in what the correct human body posture consists of

**Upper limb and head positioning in photographic examination** The hair is tied with the use of a hair clip to make the external auditory meatus and the upper body contours visible. Children are asked to look forward at eye level. For the front and back photos, the upper limbs are loosely hanging down. For the lateral photos, in order to uncover the contour of the back, the upper limbs are slightly flexed in the gleno-humeral and the elbow joint at the angle of approx. 10°–20° and 20°–30° respectively. The gleno-humeral joint flexion is performed slowly to avoid any trunk movement, especially the backward trunk hyperextension (Fig. 5). For the front photos taken during forward bending, the upper limbs are kept together and directed forward to the ground as in Adam’s test (Fig. 3). For lateral photos made during forward bending, the upper limbs are loosely hanging down (Fig. 2d, h).

#### Photographic parameters for the frontal plane evaluation

There are two main photographic parameters for the frontal plane trunk assessment and two for the lower limb assessment. The two trunk parameters are Anterior Trunk Symmetry Index and Posterior Trunk Symmetry Index.

**Fig. 6:**
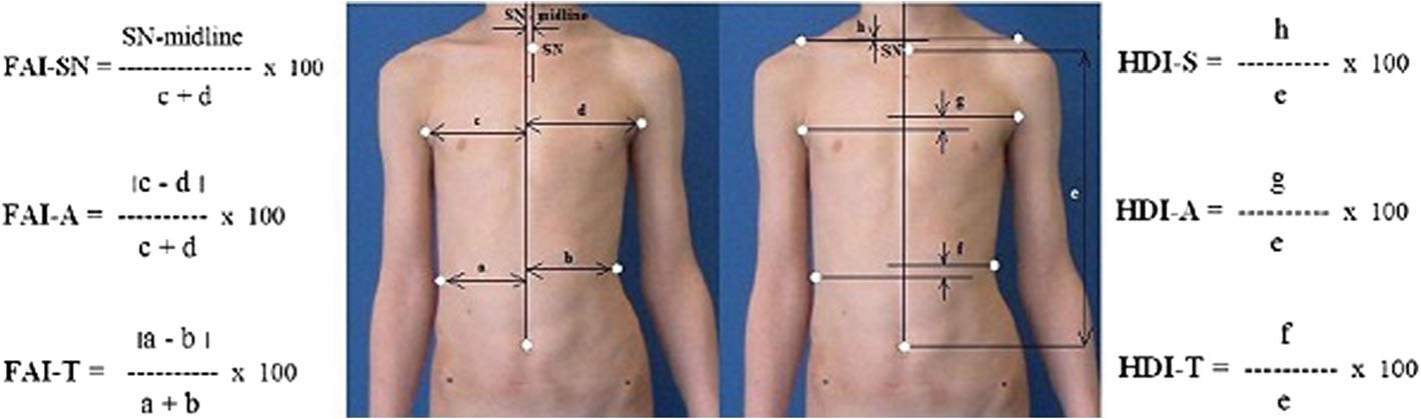
Diagram illustrating the measurements of ATSI Index

Anterior Trunk Symmetry Index (ATSI)—the parameter is defined as the sum of six indices: three frontal plane asymmetry indices (sternal notch, axilla folds, and waist lines) and three frontal plane height difference indices (acromions, axilla folds, and waist lines). Frontal asymmetry index at sternal notch level (FAI-SN) is calculated by dividing the distance between the center of the sternal notch and the midline by the height of the trunk. The height of the trunk (e) is the vertical distance between the navel and the center of the sternal notch. Frontal asymmetry indexes at axilla level (FAI-A) and at trunk level (FAI-T) are calculated by dividing the difference in the distance between each trunk’s edge and the midline (c − d, a − b) by the width of the trunk (c + d, a + b). Height indices of trunk asymmetry are calculated by dividing the difference in height at three levels of trunk: HDI-S for shoulders, HDI-A for axillas, and HDI-T for the trunk waistline by the trunk height measured from navel to the center of the sternal notch (e). The shoulder point is the point of intersection at shoulder level with a vertical line from each axilla. ATSI was introduced by Stolinski et al. in 2012 [16] (Fig. 6).

$$ \mathrm{ATSI}=\left(\mathrm{FAI}-\mathrm{S}\mathrm{N}+\mathrm{FAI}-\mathrm{A}+\mathrm{FAI}-\mathrm{T}\ \right)+\left(\mathrm{HDI}-\mathrm{S}+\mathrm{HDI}-\mathrm{A}+\mathrm{HDI}-\mathrm{T}\right) $$

**Fig. 7:**
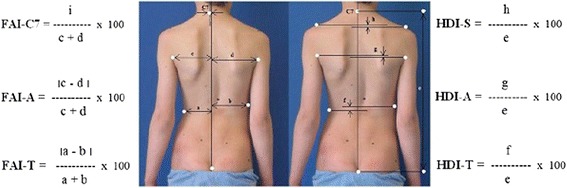
Diagram illustrating the measurements of POTSI Index

Posterior Trunk Symmetry Index (POTSI)—similarly to ATSI Index, the POTSI parameter is defined as the sum of six indices: three frontal plane asymmetry indices (C7, axilla folds, and waist lines) and three frontal plane height difference indices (acromions, axilla folds, and waist lines). Frontal asymmetry index at C7 level (FAI-C7) is calculated by dividing the distance between the C7 point and the midline by the height of the trunk. The height of the trunk (e) is the vertical distance between the C7 and the beginning of gluteal cleft. Frontal asymmetry indexes at axilla level (FAI-A) and trunk level (FAI-T) are calculated by dividing the difference in distance between each trunk’s edge and the midline (c − d, a − b) by the width of the trunk (c + d, a + b). Height indices of trunk asymmetry are calculated by dividing the difference in the height at three levels of trunk: HDI-S for shoulders, HDI-A for axillas, and HDI-T for the trunk waistline by the trunk height (e). The shoulder point is the point of intersection at shoulder level with a vertical line from each axilla. POTSI was introduced by Suzuki et al. in 1999 [17, 18] (Fig. 7).

$$ \mathrm{POTSI}=\left(\mathrm{FAI}-\mathrm{C}7+\mathrm{FAI}-\mathrm{A}+\mathrm{FAI}-\mathrm{T}\ \right)+\left(\mathrm{HDI}-\mathrm{S}+\mathrm{HDI}-\mathrm{A}+\mathrm{HDI}-\mathrm{T}\right) $$

The two photographic postural parameters of lower limb frontal plane assessment are tibiofemoral angle and tibiocalcaneal angle.

**Fig. 8:**
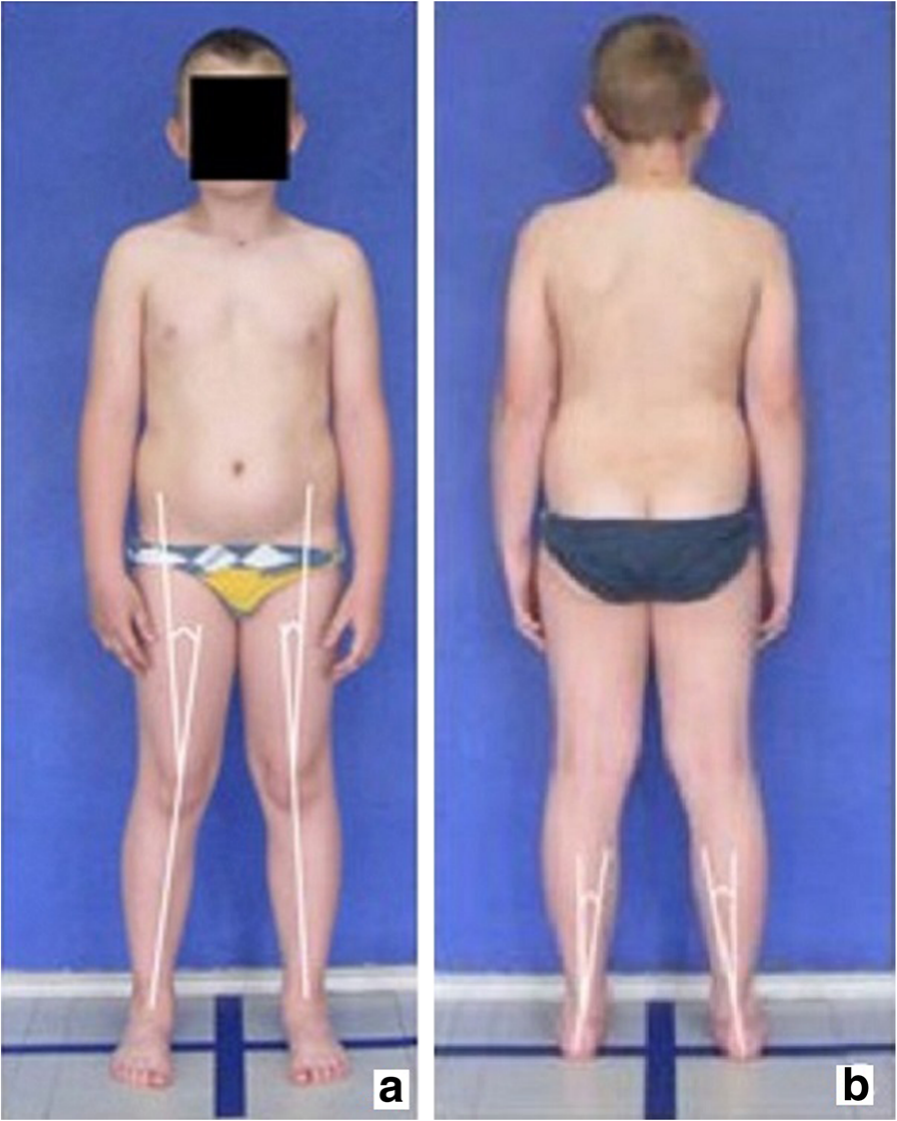
**a** Diagram illustrating the measurements of TFA. **b** Diagram illustrating the measurements of TCA

Tibiofemoral angle (TFA)—the angle between the line drawn from the center of the ankle joint to the center of the knee joint and the line drawn from the center of the knee joint to ASIS of the same lower limb (Fig. 8a) [19, 20].

Tibiocalcaneal angle (TCA)—the angle between a line drawn between the center of the calcaneus and the Achilles tendon, and a second line drawn from the Achilles tendon to the mid-calf of the same lower limb (Fig. 8b) [21].

#### Photographic parameters for the sagittal plane evaluation

**Fig. 9:**
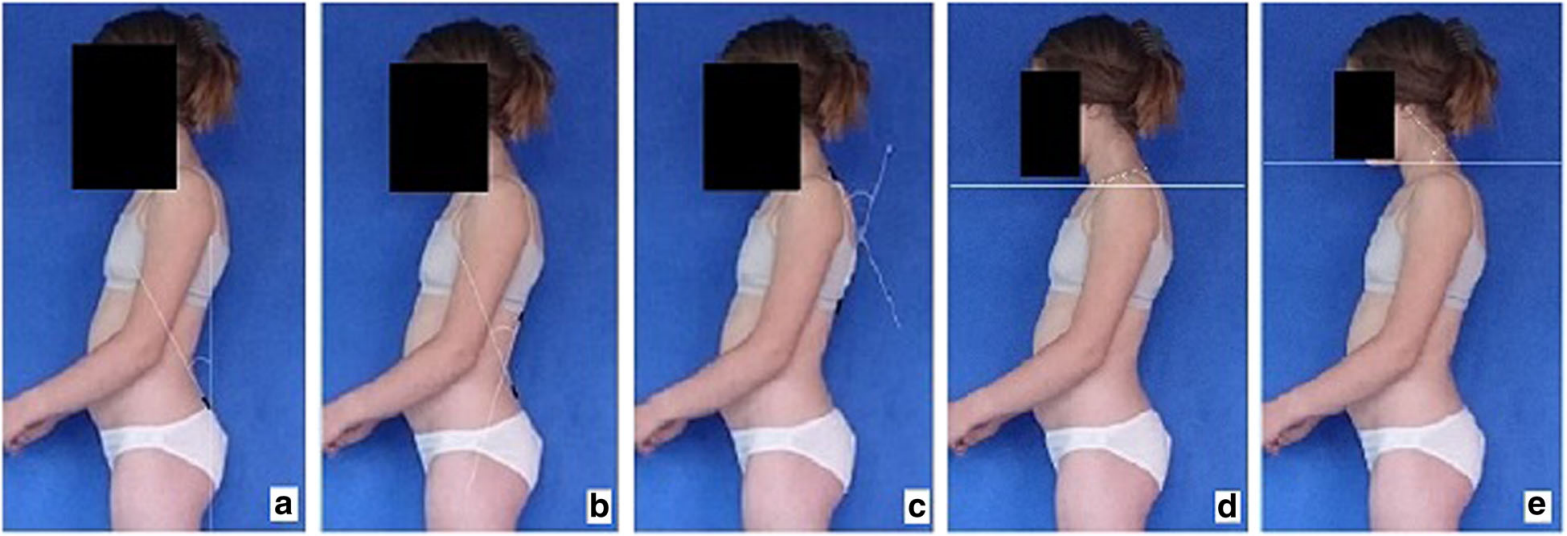
**a** Diagram illustrating the measurements of SS. **b** Diagram illustrating the measurements of LL. **c** Diagram illustrating the measurements of TK. **d** Diagram illustrating the measurements of CI. **e** Diagram illustrating the measurements of HP

**Fig. 10:**
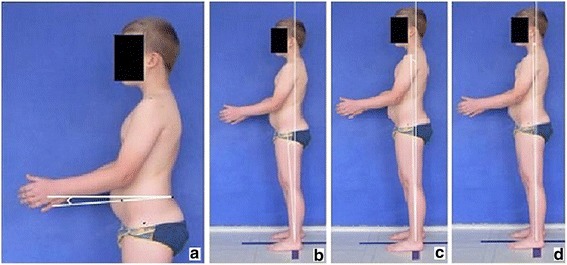
**a** Diagram illustrating the measurements of SPT. **b** Diagram illustrating the measurements of TA. **c** Diagram illustrating the measurements of AA. **d** Diagram illustrating the measurements of EA

The following photographic parameters are assumed for the sagittal plane assessment:

- Sacral slope angle (SS)—the angle between the vertical line and the line tangent to body contour at the sacral area (Fig. 9a) [22].
- Lumbar lordosis angle (LL)—the angle between the line tangent to body contour at the level of T12-L1 spinous processes and the line tangent to body contour at the level of L5-S1 spinous processes (Fig. 9b) [23].
- Thoracic kyphosis angle (TK)—the angle between the line tangent to body contour at the level of C7-Th1 spinous processes and the line tangent to body contour at the level of Th12–L1 spinous processes (Fig. 9c) [24].
- Chest inclination angle (CI)—the angle between the horizontal line and the line connecting the C7 spinous process with the point at the anterior neck-anterior thorax junction (Fig. 9d) [25].
- Head protraction angle (HP)—the angle between the horizontal line and the line connecting the C7 spinous process and the external auditory meatus (Fig. 9e) [26].
- Sagittal pelvic tilt (SPT)—the angle between the horizontal line and the line joining the anterior and the posterior superior iliac spine (Fig. 10a) [27].
- Trochanter-ankle angle (TA)—the angle between the vertical line drawn from the center of external malleolus of the ankle joint and the line drawn from the center of external malleolus of the ankle joint to the top of the greater trochanter (Fig. 10b).
- Acromion-ankle angle (AA)—the angle between the vertical line drawn from the center of external malleolus of the ankle joint and the line drawn from the center of external malleolus of the ankle joint to the center of acromion (Fig. 10c).
- Ear-ankle angle (EA)—the angle between the vertical line drawn from the center of external malleolus of the ankle joint and the line drawn from the center of external malleolus of the ankle joint to the external auditory meatus (Fig. 10d).
- Enlarged photos of coronal and sagittal parameters are presented in Additional file 1: Appendix 1.

### Semi-automatic measurements of postural photographic parameters

**Fig. 11:**
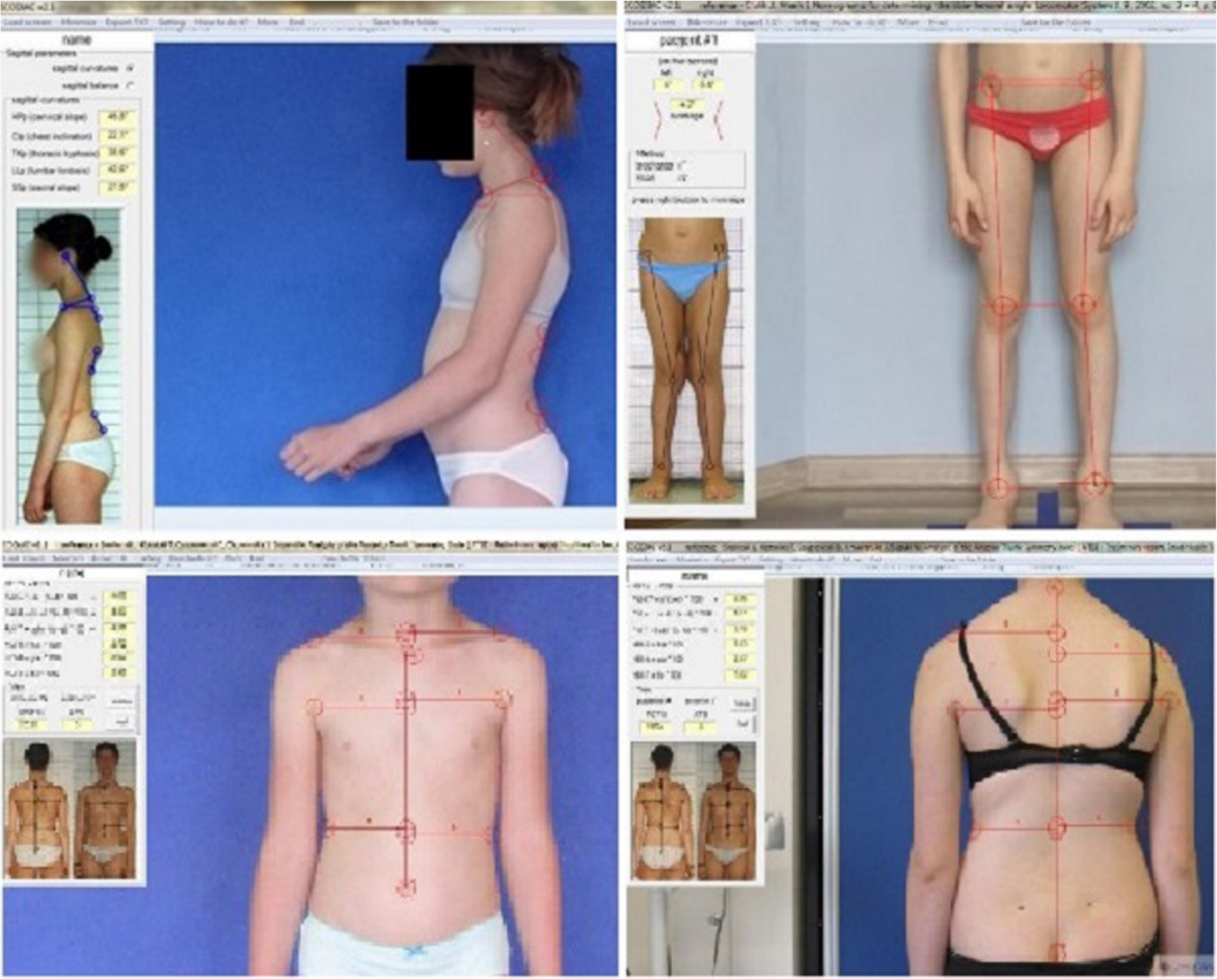
SCODIAC printscreen images illustrating coronal and sagittal plane parameters

All the abovementioned parameters can be measured manually, manually in ink, on a print or digitally on the monitor screen. To facilitate the measurement, a semi-automatic software named SCODIAC was created [28]. The software is available online and free to download [https://www.ortotika.cz/download/SetupSCODIAC_Full.zip]. The landmarks are manually placed on the screen. Afterwards, the software calculates the values of the required parameters. The initial version of software was checked against x-ray measurements [29]. The current version focused on digital photography images (Fig. 11). Placing the landmarks consists in moving small circles provided at the screen to the required anatomical points manually. The software calculations are automatic. The software explains all functions in a user-friendly way.

### Validation of the photographic technique

We checked the reliability of the photographic technique above. Our objectives in this part of the study were (1) to determine the intra-observer reproducibility and the inter-observer reliability of the photographic sagittal parameters: sacral slope angle (SS), lumbar lordosis angle (LL), thoracic kyphosis angle (TK), chest inclination angle (CI), and head protraction angle (HP) (Fig. 9); and (2) to check the validity of photographic measurements against the Rippstein plurimeter measurements by analyzing correlations between the corresponding angles.

The study group consisted of 91 healthy volunteers (44 girls and 47 boys) aged 7–10 (mean 8.2 ± 1.0 years). The exclusion criteria were history of any spine disorder, min. 7-degree ATR value, lower limbs discrepancy, and refusal to participate. Children were photographed in a relaxed (spontaneous, habitual) posture from the left (Fig. 2b) and right side (Fig. 2f). The study was performed in accordance with the 1964 Helsinki Declaration. All studies reported in this chapter were approved by the Institutional Review Board of Poznan University of Medical Sciences (No. 832/11, date 6/10/2011).

#### Intra-observer reproducibility

One observer (a physiotherapist with 10 years’ experience) performed three series of photographic measurements. Each series comprised three measurements, with a 2-day interval between each series. The observer measured the photographic parameters of 30 randomly selected healthy children. Five photographic parameters (SS, LL, TK, CI, and HP) were measured using the aforementioned methodology. The intra-observer reproducibility was quantified by the use of intraclass correlation coefficient (ICC) and standard error for single measurement (SEM) [30].

#### Inter-observer reliability

Three observers, physiotherapists with 10, 8, and 2 years’ experience respectively, performed three series of photographic measurements. Each series included three measurements, with a 2-day interval between each series. The observer measured photographic parameters of 30 randomly selected healthy children. Five photographic parameters (SS, LL, TK, CI, and HP) were measured using the methodology described above. The inter-observer reliability was quantified by the use of intraclass correlation coefficient (ICC) and standard error for single measurement (SEM) [30].

#### Validation of the photographic technique against Rippstein plurimeter

**Fig. 12:**
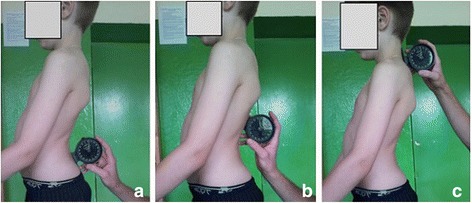
Areas of application of the Rippstein plurimeter to the patient’s spine: **a** lumbo-sacral junction, **b** thoraco-lumbar junction, and **c** cervico-thoracic junction. The angular parameters are calculated as the differences between the two positions: lumbar lordosis = **a**, **b**; thoracic kyphosis = **b**, **c**

In order to determine the correlation of the photographic parameters versus Rippstein plurimeter measurements, three observers measured the sagittal curvatures (sacral slope, lumbar lordosis, and thoracic kyphosis) of 91 children three times with the use of the Rippstein plurimeter (Fig. 12) immediately after the children had the photos taken, one photo from the left side and one photo from the right side, according to standardized conditions described above. The values of the corresponding parameters (photographic thoracic kyphosis angle versus plurimeter thoracic kyphosis angle, etc.) were compared.

### Variability of photographic sagittal parameters over time

The aim of the second part of the study was to analyze the variability over time (zero time, after 1 h, and after 1 week) of five 2D photographic angles: sacral slope (SS), lumbar lordosis (LL), thoracic kyphosis (TK), chest inclination (CI), and head protraction (HP).

The study group comprised 30 healthy volunteers (13 girls and 17 boys) aged 7–10 (mean 8.2 ± 1.0 years). The same exclusion criteria as in photographic technique validation XYZ were used. Children were photographed in a standardized relaxed (spontaneous, habitual) posture (Fig. 2b). At each of the three exposures, the digital photographs of the left profile of the body were taken three times one after another within 5 s. The exposure was made (1) at the time zero, (2) 1 h later, and (3) one week later. In total, 270 photos were assessed. Five photographic parameters were calculated on each photo.

### Variability of photographic coronal parameters over time

The aim of this part of the study was to analyze the variability in time (zero time, after one hour, after one week) of two coronal photographic parameters: ATSI (Anterior Trunk Symmetry Index) (Fig. 6) and POTSI (Posterior Trunk Symmetry Index) (Fig. 7) which serve to evaluate the symmetry of the trunk in coronal plane.

**Fig. 13:**
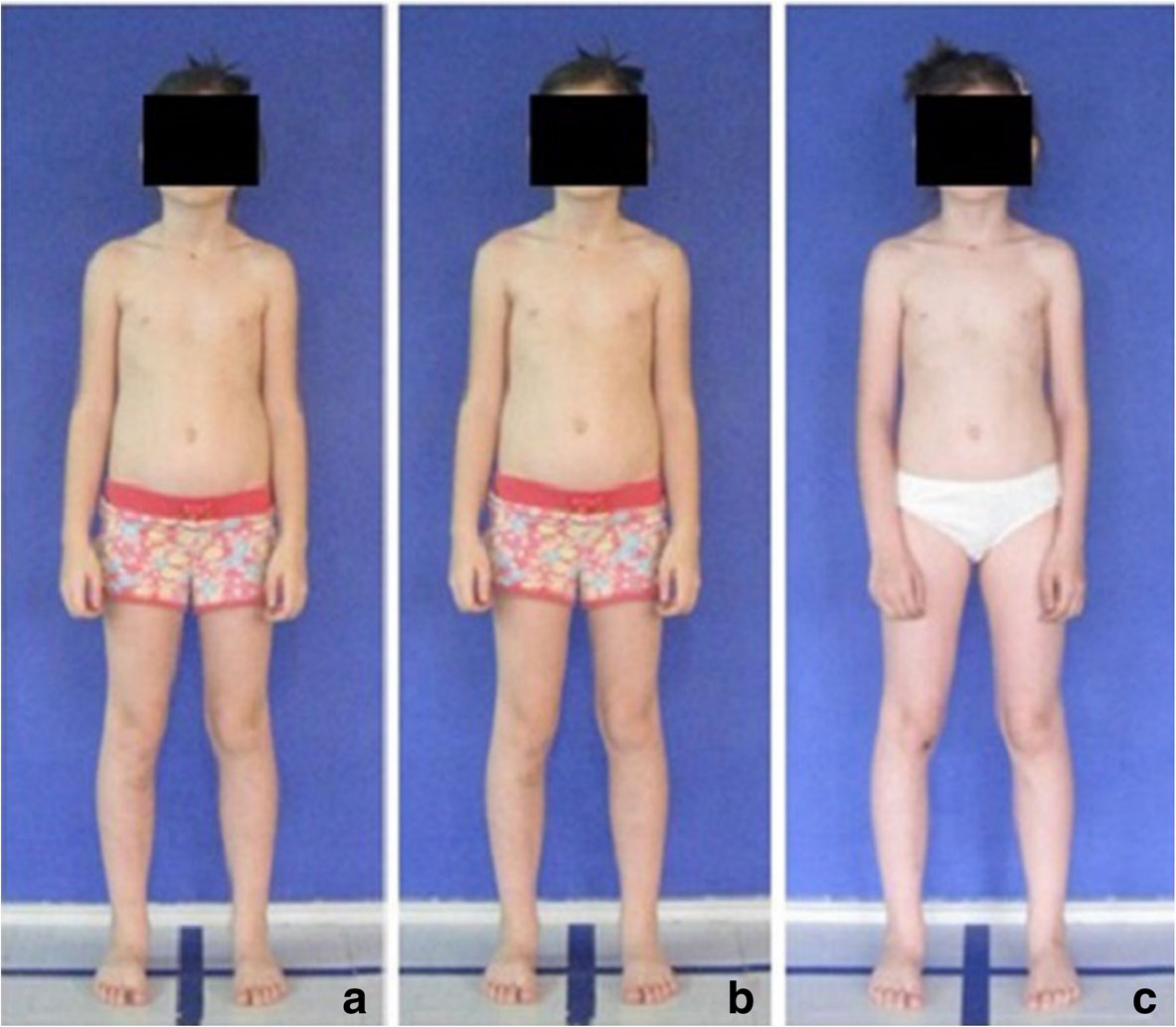
Positioning of the child during photographic documentation of front view: **a** zero time, **b** after 1 h, and **c** after 1 week

**Fig. 14:**
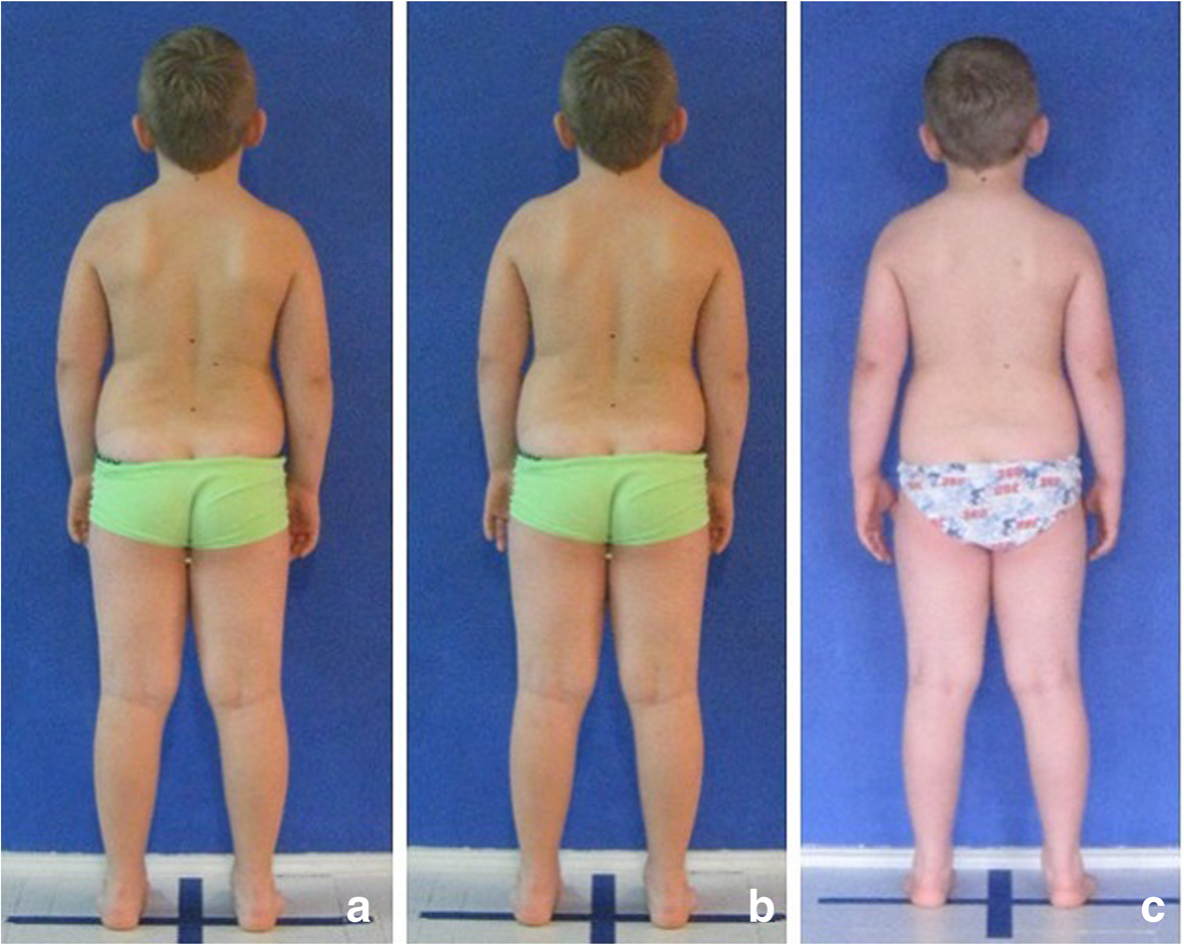
Positioning of the child during photographic documentation of back view: **a** zero time, **b** after 1 h, and **c** after 1 week

The study group comprised 30 healthy volunteers (13 girls and 17 boys) aged 7–10 (mean 8.1 ± 1.1 years). The same exclusion criteria as in photographic technique validation were used. Children were photographed in a standardized relaxed (spontaneous, habitual) posture in the coronal plane. Three digital photographs were taken within 5 s, including the front (Fig. 13) and back (Fig. 14) view. The same procedure was repeated after 1 h and after 1 week (540 photos were assessed).

### Normative values of sagittal photographic parameters in children aged 7–10

Normative values of sagittal photographic parameters were calculated based on photographic assessment of 7782 children of both sexes, aged 7–10. All photographs were taken respecting the abovementioned procedures.

### Statistical analysis

Statistical analyses were performed using Statistica 10 (StatSoft), Gretl and Microsoft Excel software. Statistical significance level was defined as *P <* 0.05. Reliability was determined with the intraclass correlation coefficient (ICC) by means of the two-way model and Cronbach’s alpha. [30, 31]. The scale from Bland and Altman were used in the classification of the reliability values and relationship between plurimeter and photography [30]. ICC values smaller than or equal to 0.20 were considered poor, 0.21–0.40 fair, 0.41–0.60 moderate, 0.61–0.80 good, and 0.81–1 very good [32]. Standard error of measurement (SEM) was measured according to Shrout. [33]. Analysis of variance, homogeneity of variance, normality of distribution, and post hoc tests were used to examine the variation of five photographic sagittal parameters over time.

## Results

### Photogrammetry reliability studies

#### Validation of the photographic technique

##### Photographic measurements

The reliability of the photographic measurements is shown in Table 1. The ICC values for the sacral slope angle, lumbar lordosis angle, thoracic kyphosis angle, chest inclination angle, and head protraction angle revealed very good reliability, with the SEMs of the measurement ranging between 0.7 and 1.3.

**Table 1:** Reliability of using photographic technique for measuring the sagittal trunk alignment

Variables	Intraobserver reproducibility					Interobserver reliability				
	ICC	95%	CI	SEM [°]	*P* value	ICC	95%	CI	SEM [°]	*P* value
Left side of the body										
SS	0.93	0.88	0.97	1.0	0.957	0.93	0.86	0.97	0.9	0.658
LL	0.97	0.95	0.99	1.0	0.975	0.97	0.95	0.99	1.0	0.987
TK	0.93	0.87	0.96	1.2	0.974	0.94	0.89	0.97	0.9	0.811
CI	0.96	0.92	0.98	0.7	0.953	0.92	0.83	0.96	0.9	0.540
HP	0.90	0.83	0.95	1.0	0.990	0.84	0.74	0.92	1.2	0.984
Right side of the body										
SS	0.93	0.88	0.97	1.0	0.952	0.92	0.86	0.96	1.1	0.954
LL	0.96	0.93	0.98	1.2	0.936	0.96	0.92	0.98	1.1	0.852
TK	0.91	0.85	0.95	1.3	0.990	0.92	0.86	0.96	1.2	0.726
CI	0.93	0.87	0.96	0.9	0.931	0.88	0.79	0.94	1.1	0.689
HP	0.94	0.89	0.97	0.7	0.989	0.85	0.75	0.92	1.0	0.913
Mean of the left and right side of the body										
SS	0.95	0.91	0.97	0.9	0.977	0.94	0.89	0.97	0.9	0.836
LL	0.97	0.95	0.98	1.0	0.952	0.98	0.95	0.99	0.9	0.936
TK	0.93	0.88	0.97	1.1	0.986	0.92	0.86	0.96	0.9	0.777
CI	0.96	0.92	0.98	0.7	0.950	0.92	0.84	0.96	0.9	0.622
HP	0.94	0.89	0.97	0.8	0.995	0.89	0.80	0.94	1.0	0.979

*ICC* intraclass correlation coefficient, *CI* confidence interval, *SEM* standard error of measurement

*Statistically significant difference (*P* < .05)

##### Photogrammetry versus plurimeter

The correlation of measurements using plurimeter and digital photography is shown in Table 2. The ICC values for the sacral slope angle (0.93), lumbar lordosis angle (0.97), and thoracic kyphosis angle (0.95) revealed very good reliability. All ICC values for the three angles reported very good interobserver repeatability, with the SEMs of the measurement ranging between 0.9 and 1.4.

**Table 2:** Correlation of Rippstein plurimeter versus photographic measurements

Variables	ICC	95%	CI	SEM [°]	*P* value
Plurimeter SS—photo SS	0.93	0.89	0.95	1.2	0.712
Plurimeter LL—photo LL	0.97	0.93	0.98	0.9	0.425
Plurimeter TK—photo TK	0.95	0.93	0.97	1.4	0.945

*ICC* intraclass correlation coefficient, *CI* confidence interval, *SEM* standard error of measurement

*Statistically significant difference (*P* < .05)

##### Variability of photographic sagittal parameters over time

There were no significant differences between the measurements (*p* > 0.05) at zero time, after 1 h, and after 1 week in any of the five sagittal photographic parameters. In the case of SS and CI, the 1 week measurement was different to the zero and the 1-h measurement, but the differences were not statistically significant (using analysis of variance and post hoc tests). The results of measurement of both parameters increased with time, so the largest difference was observed between the measurement carried out in time zero and 1 week later. In case of the remaining three parameters (TK, LL, HP), we could not find such a trend (Table 3).

**Table 3:** Variability of sagittal and frontal parameters over time

Measurements	SS		LL		TK		CI		HP		ATSI		POTSI	
	Mean	SD	Mean	SD	Mean	SD	Mean	SD	Mean	SD	Mean	SD	Mean	SD
Zero time	24.7	7.0	41.7	9.5	44.5	6.7	27.1	7.1	53.3	3.1	21.6	11.6	21.3	10.0
After 1 h	25.3	7.2	42.0	8.3	43.6	7.6	27.6	7.5	52.8	5.8	21.8	11.4	21.9	10.6
After 1 week	26.8	8.1	43.0	9.1	45.0	8.9	29.3	6.8	52.8	5.1	20.1	8.8	18.3	6.1
*P*	0.533		0.854		0.786		0.478		0.926		0.798		0.288	

*Mean* mean value of three measurements, *SD* standard deviation of three measurements

*Statistically significant difference (*P* < .05)

##### Variability of photographic coronal parameters over time

There was no statistically significant difference between measurements (*p* > 0.05) for ATSI in zero time, after 1 h, and after 1 week. There was no statistically significant difference between measurements (*p* > 0.05) for POTSI parameters in zero time, after 1 h, and after 1 week (Table 4). A slight tendency regarding the difference between the 1-week measurement and the zero and 1-h measurement was not statistically significant. This observation needs further study in a bigger sample (*p* values in post hoc tests were between 0.15 and 0.30).

**Table 4:** Exemplary table based on numerical values for 7-year-old girls (*N* = 1083)

Percentile	SS	LL	TK	CI	HP
97	44	52	62	41	71
90	39	45	55	36	66
75	33	37	48	32	63
*50*	*27*	*29*	*42*	*27*	*58*
25	22	23	35	22	54
10	17	18	28	18	49
3	12	13	23	14	45

##### Normative values of sagittal photographic parameters for children 7–10

Five sagittal photographic parameters (SS, LL, TK, CI, HP) were measured for each child. The data was analyzed separately for boys and girls and for each year of age, ranging from 7 to 10. Numerical values based on the tables (Additional file 2: Appendix 2A) and percentile charts for sex and age (Additional file 2: Appendix 2B) are presented in Additional file 2: Appendix 2. Table 4 contains the exemplary numerical values of the five photographic parameters (all values presented in degrees).

##### Pitfalls and sources of errors in photogrammetry used for posture evaluation

Errors may occur during photographic examination and photography evaluation. Attention should be paid to prepare and position the child according to the protocol. The incorrect preparation or positioning is illustrated below with the examples identified within our study group of 7782 children participating in the local school screening program. In total, 46,595 digital photos were analyzed.

**Fig. 15:**
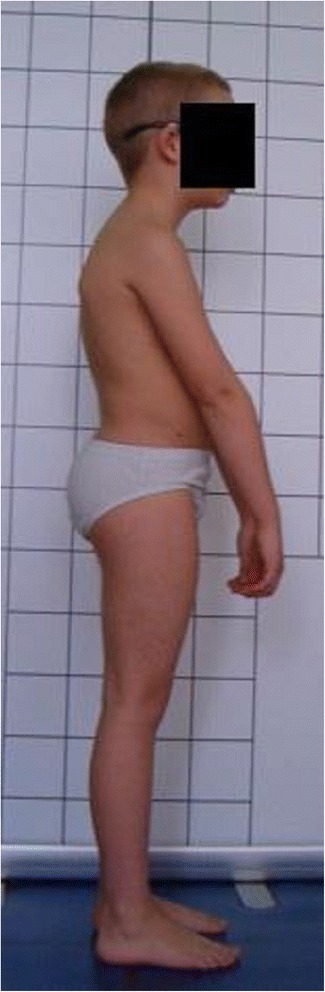
Technical error—protraction of the shoulders

**Fig. 16:**
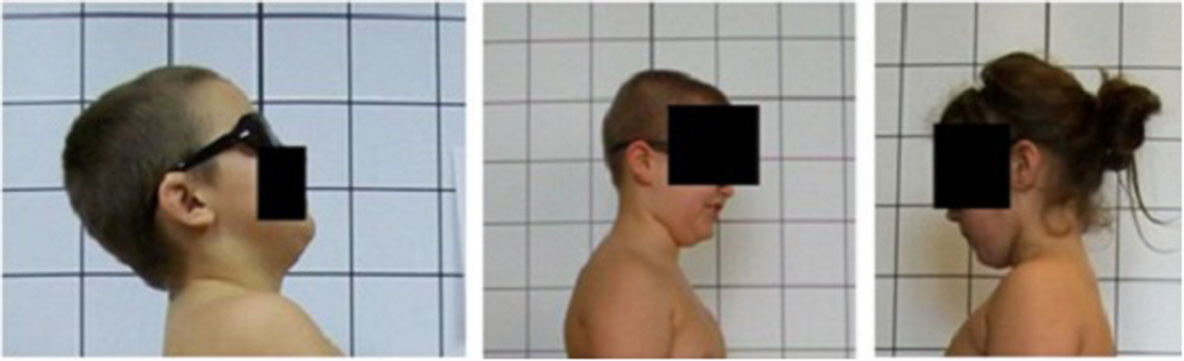
Technical error—incorrect head position and/or gaze direction

**Fig. 17:**
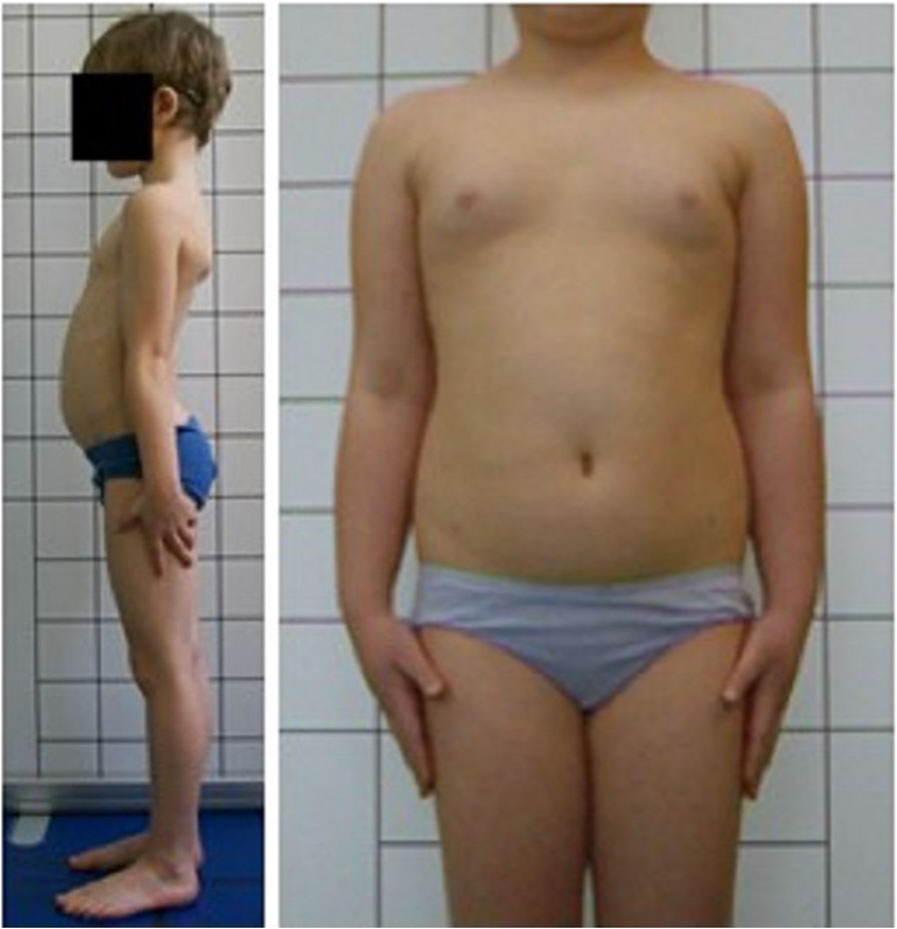
Technical error—lack of spontaneous relaxed posture

**Fig. 18:**
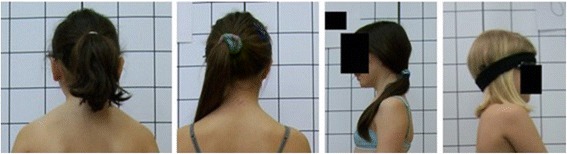
Technical error—body contours covered by hair

**Fig. 19:**
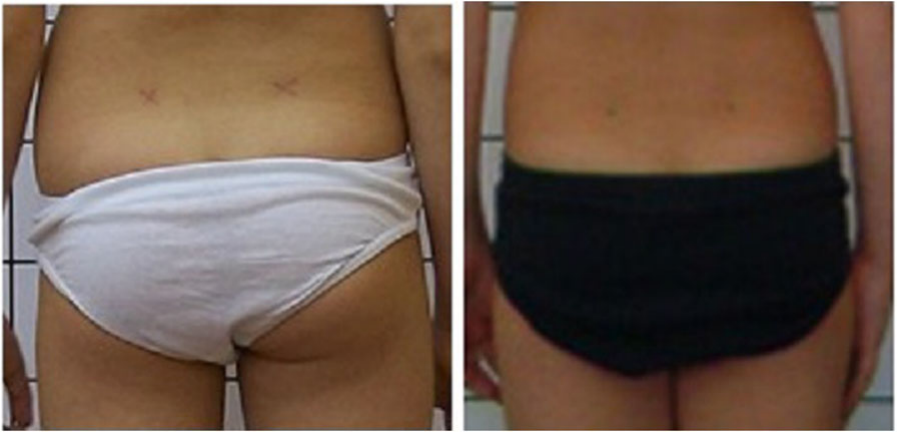
Technical error—gluteal cleft upper contour covered by underpants

**Fig. 20:**
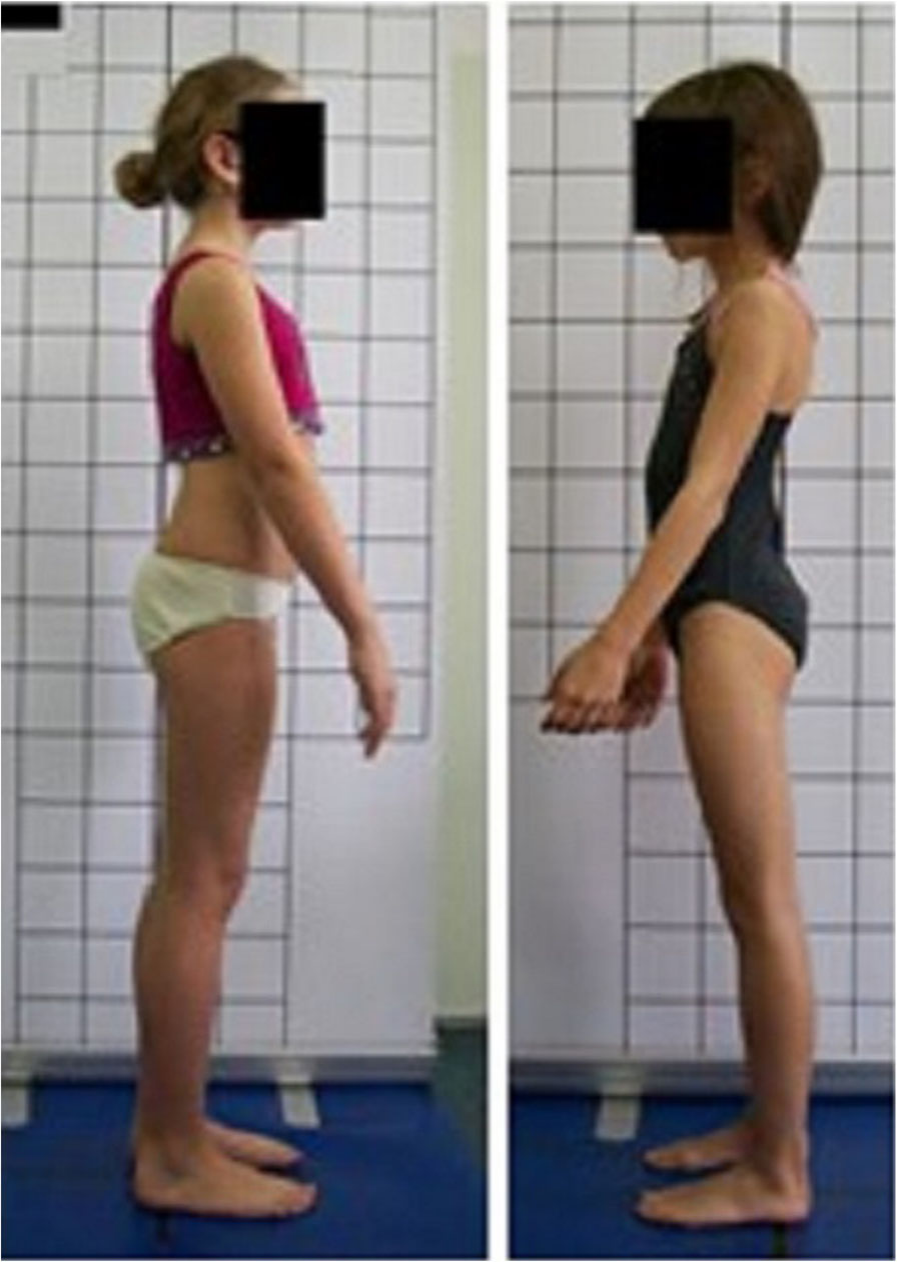
Technical error—the trunk obscured by bra or swimsuit

**Fig. 21:**
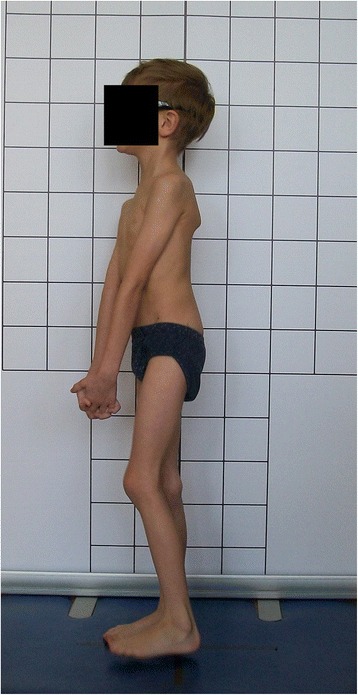
Technical error—one-leg standing

**Fig. 22:**
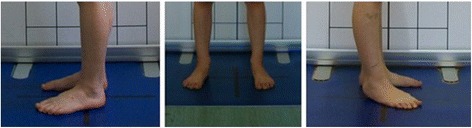
Technical error—incorrect feet position unparalleled

**Fig. 23:**
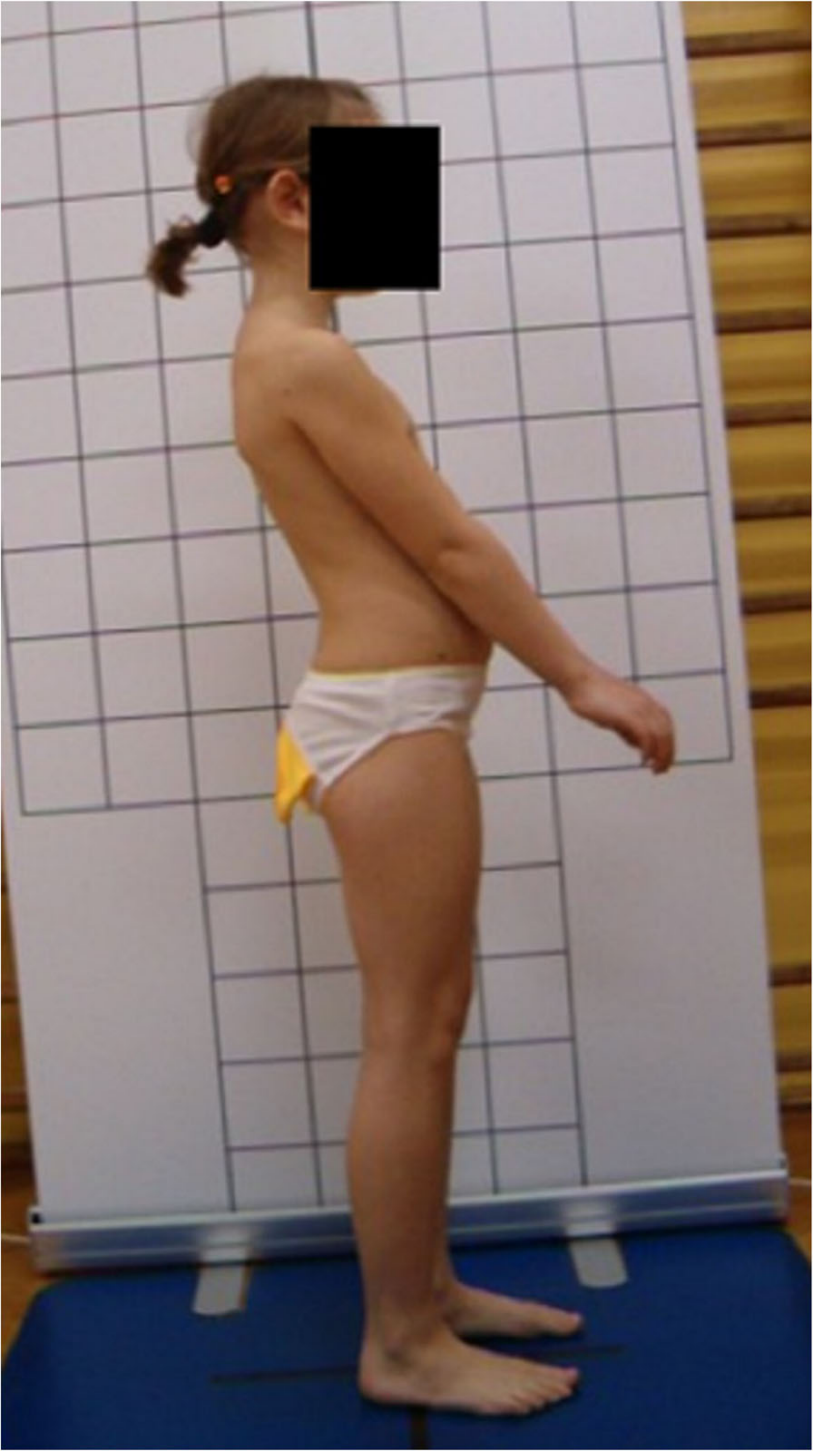
Technical error—digital camera not leveled

**Fig. 24:**
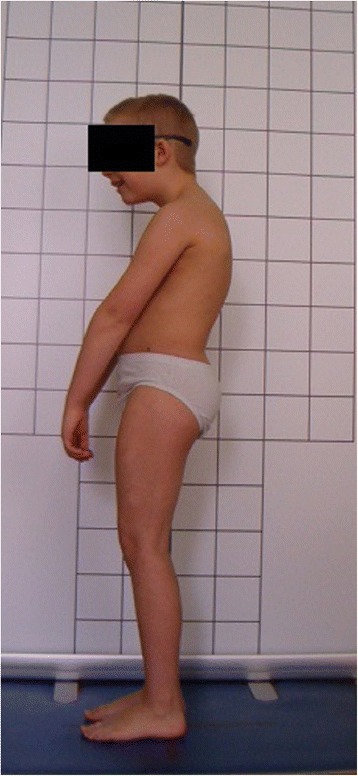
Technical error—limited communication with child

**Fig. 25:**
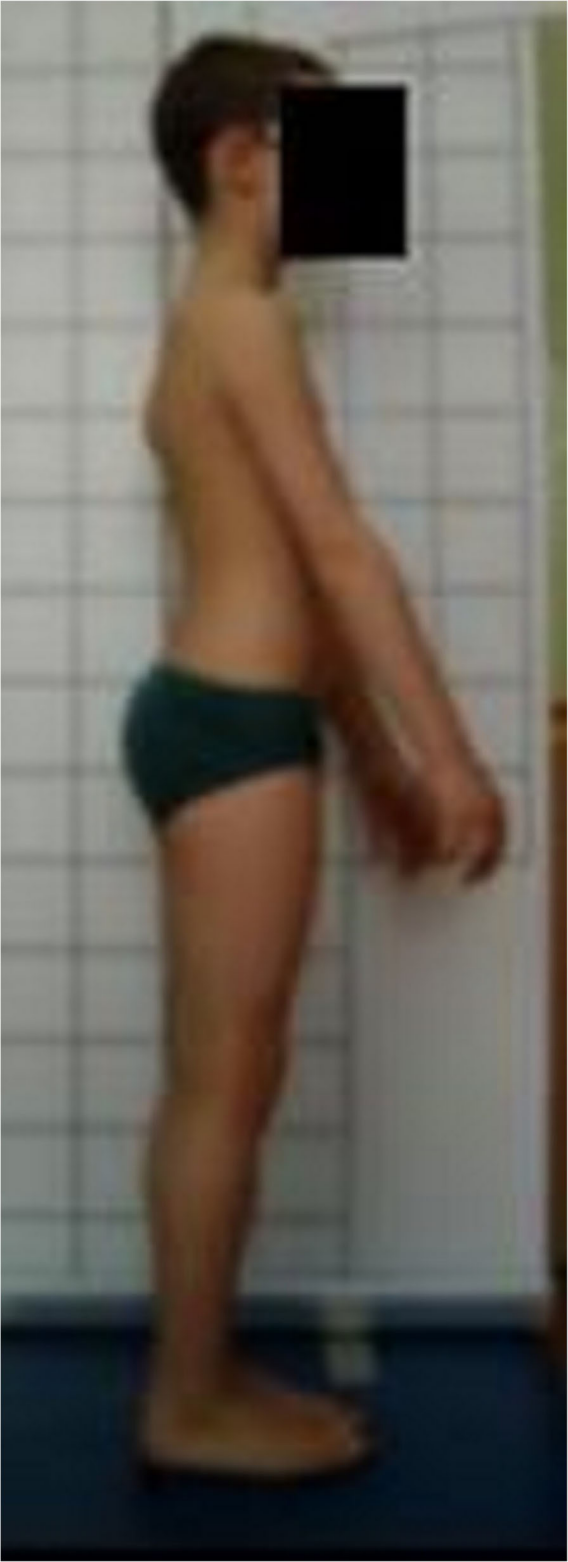
Technical error—photo taken without proper focusing

The following problems were noted and are reported below in the following way: (1) type of error and (2) consequence for posture assessment. Figures are illustrating the following:

- Protraction of the shoulders—the upper limbs cover the body contours and anatomical points (Fig. 15)
- Incorrect head position and gaze direction—impact on cervical spine parameters (Fig. 16)
- Inability to adopt spontaneous relaxed posture—impact on lumbar lordosis and thoracic kyphosis angles (Fig. 17)
- Hair covering the body contours—impossibility of measuring photographic parameters (Fig. 18)
- Gluteal cleft covered with underpants—impossible calculation of POTSI index (Fig. 19)
- Bra or swimsuit with limited body contact and obscuring the trunk—sagittal angles design and calculation not possible (Fig. 20)
- One-leg standing—impact on coronal plane symmetry (Fig. 21)
- Incorrect rotational foot positioning—introduction of rotation to the whole body (Fig. 22)
- Digital camera not level—possible photographic parameters modification (Fig. 23)
- Limited communication with the child can be treated as a contraindication for photographical measurements—standardized position not possible (Fig. 24)
- Insufficient image sharpness—difficulties with photographic angle measurement (Fig. 25)

These errors can influence the photographic evaluation and should be avoided.

## Discussion

### Radiological assessment as the current gold standard for scoliosis evaluation but not for child body posture evaluation

The radiological imaging remains the gold standard for idiopathic scoliosis (IS) diagnosis and evaluation [34–37]. It enables the primary and secondary curves identification, Cobb angle measurement, axial vertebral rotation assessment, and Risser sign grading. It differentiates the idiopathic scoliosis from the congenital one. However, for the large cohort studies or for the school screening purpose, the children are not exposed to radiography because of the radiation risk [38, 39]. In the screening conditions, the suspicion of idiopathic scoliosis is detected with manual anthropometric devices, such as the scoliometer [40–43] or smartphone with a specific device [44–46]. The basic method of school screening for idiopathic scoliosis is a clinical examination in the forward bending position (Adam’s test) with the use of scoliometer [47, 48]*.* Surface topography methods based on computerized image capturing and digitally calculated parameters are also proposed for the evaluation of patients suffering from idiopathic scoliosis. These techniques utilize raster stereography based on distortion of a grid projected onto the back [49–51] or body scanning using light beam and its distortion analysis [49, 52, 53].

Evaluation of physical deformity developing in idiopathic scoliosis presents some common areas together with the body shape evaluation in postural disorders. Similar diagnostic tools are often used. In children, it is especially important to apply the techniques which do not involve exposure to x-ray radiation. Several methods have been proposed for body posture assessment: simple photographic techniques and plumbline measures [54–57], goniometers, inclinometers and linear devices [58–60], computer-assisted methods including electrogoniometers [61], electromagnetic movement systems [62, 63], computer-assisted digitization systems [64–66], or 3D ultrasound-based motion analysis device [67]. Finally, digital photography is gaining grounds in the assessment of trunk alignment [68].

### Overview of photographic parameters proposed for posture evaluation

Photographic parameters for posture evaluation were presented by several authors. The parameters proposed in this study were selected based on the authors’ personal experience and the careful analysis of previous publications.

**Fig. 26:**
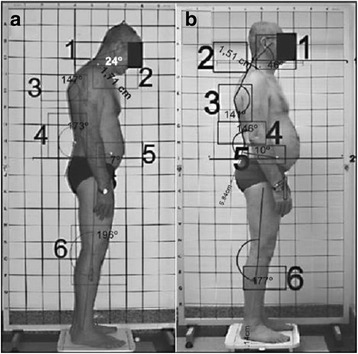
**a**–**b** Photographic assessment as proposed by Canales et al. in 2010 ([69], reprinted by permission)

Canales et al. [69] reported the following posterior and sagittal parameters: head position, thoracic kyphosis, lumbar lordosis, pelvic inclination, and knee position together with the following anatomical points to be considered: scapulas, shoulders, and ankles (Fig. 26).

**Fig. 27:**
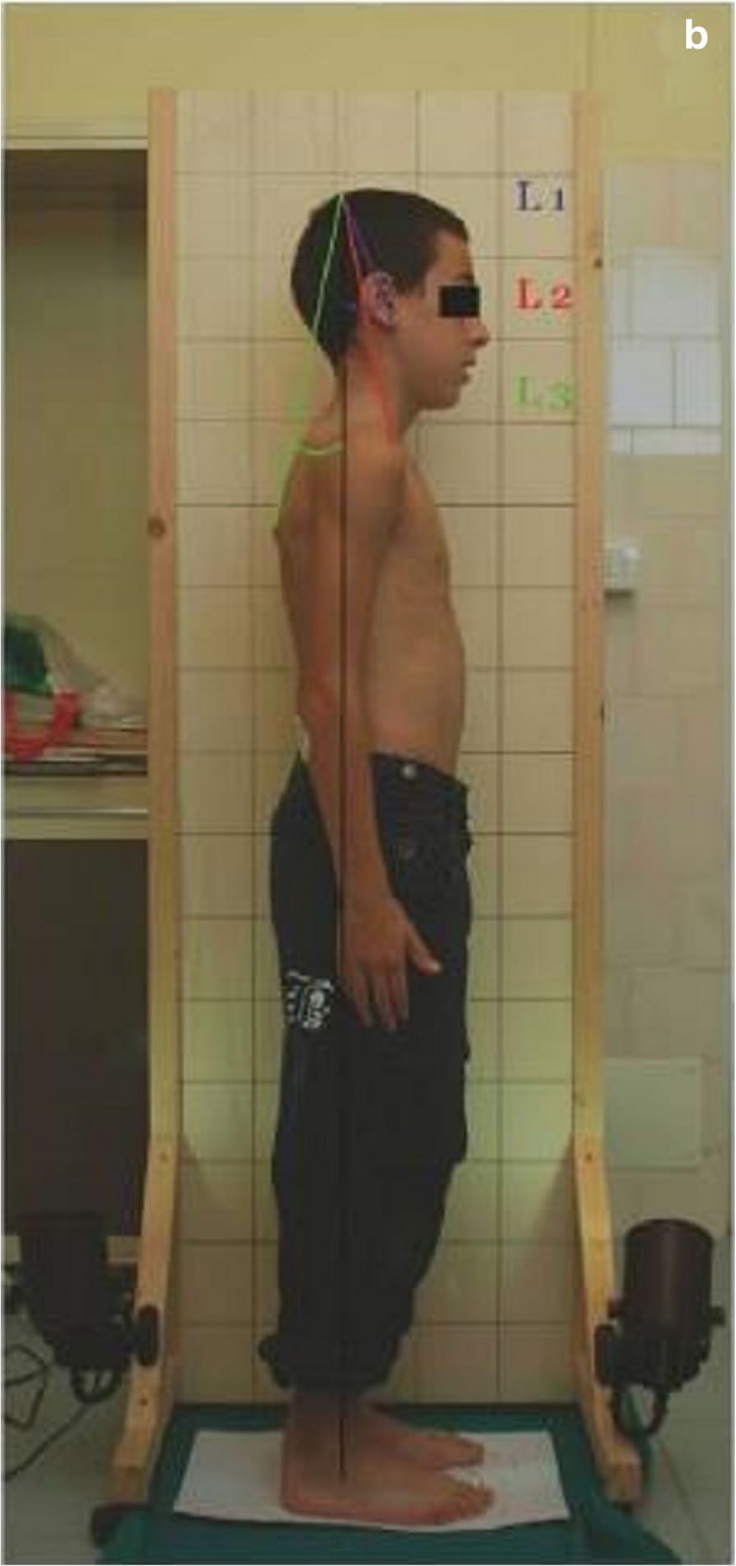
Photographic assessment as proposed by Cerutto et al. in 2012 ([70], reprinted by permission)

Cerrutto et al. [70] reported the following anterior, posterior, and sagittal parameters: P1, P2, L1, L2, L3, AR, and AL angles which were measured based on the lines drawn from the anatomical points: superior and inferior scapular angles, vertical lines related to ear lobe, acromion and scapular prominence, and vertical lines related to manubrium and coracoid process (Fig. 27).

**Fig. 28:**
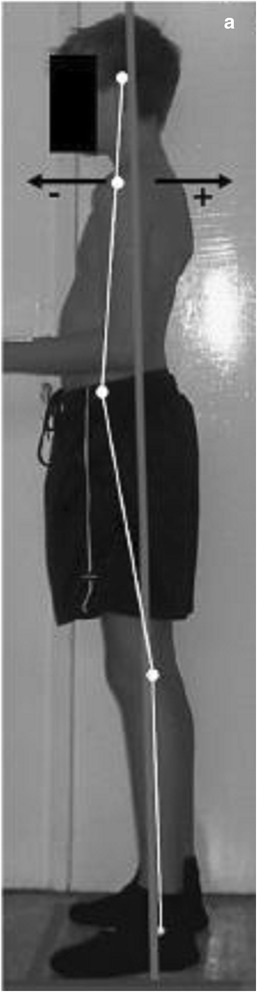
Photographic assessment as proposed by Pausić et al. in 2010 ([71], reprinted by permission)

Pausić et al. [71] proposed assessment based on the following anatomical points: head and neck, trunk, pelvis, knee joints, and ankle joints (for the coronal plane) or head and neck, trunk, pelvis, and knee joints for the sagittal plane (Fig. 28).

**Fig. 29:**
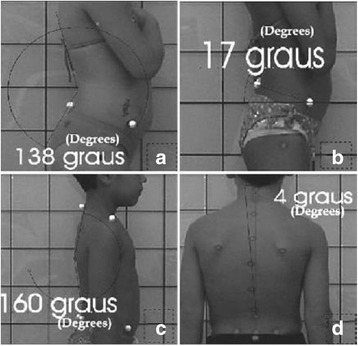
**a**–**d** Photographic assessment as proposed by Penha et al. in 2009 ([8], reprinted by permission)

Penha et al. [8] reported the following posterior and sagittal parameters: lumbar lordosis, thoracic kyphosis, pelvic inclination, head position, and lateral spinal deviations based on anatomical points to be considered (Fig. 29).

**Fig. 30:**
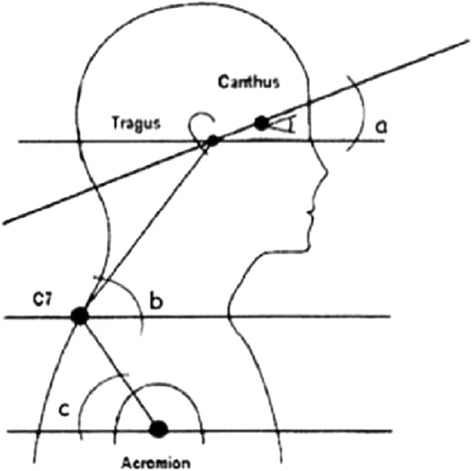
Photographic assessment as proposed by Ruivo et al. in 2015 ([72], reprinted by permission)

Ruivo et al. [72] reported the following sagittal parameters: head angle, cervical angle, and shoulder angle (Fig. 30).

**Fig. 31:**
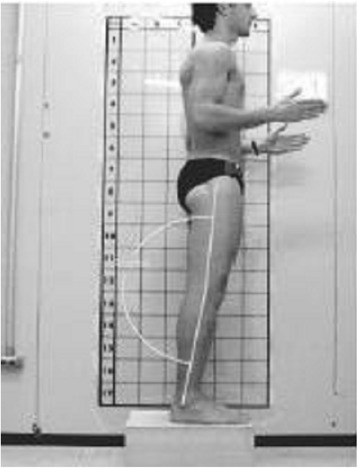
Photographic assessment as proposed by Sacco et al. in 2007 ([73], reprinted by permission)

Sacco et al. [73] reported the following sagittal parameters: tibiotarsal angle, knee extension/flexion angle, Q angle, and subtalar angle (Fig. 31).

**Fig. 32:**
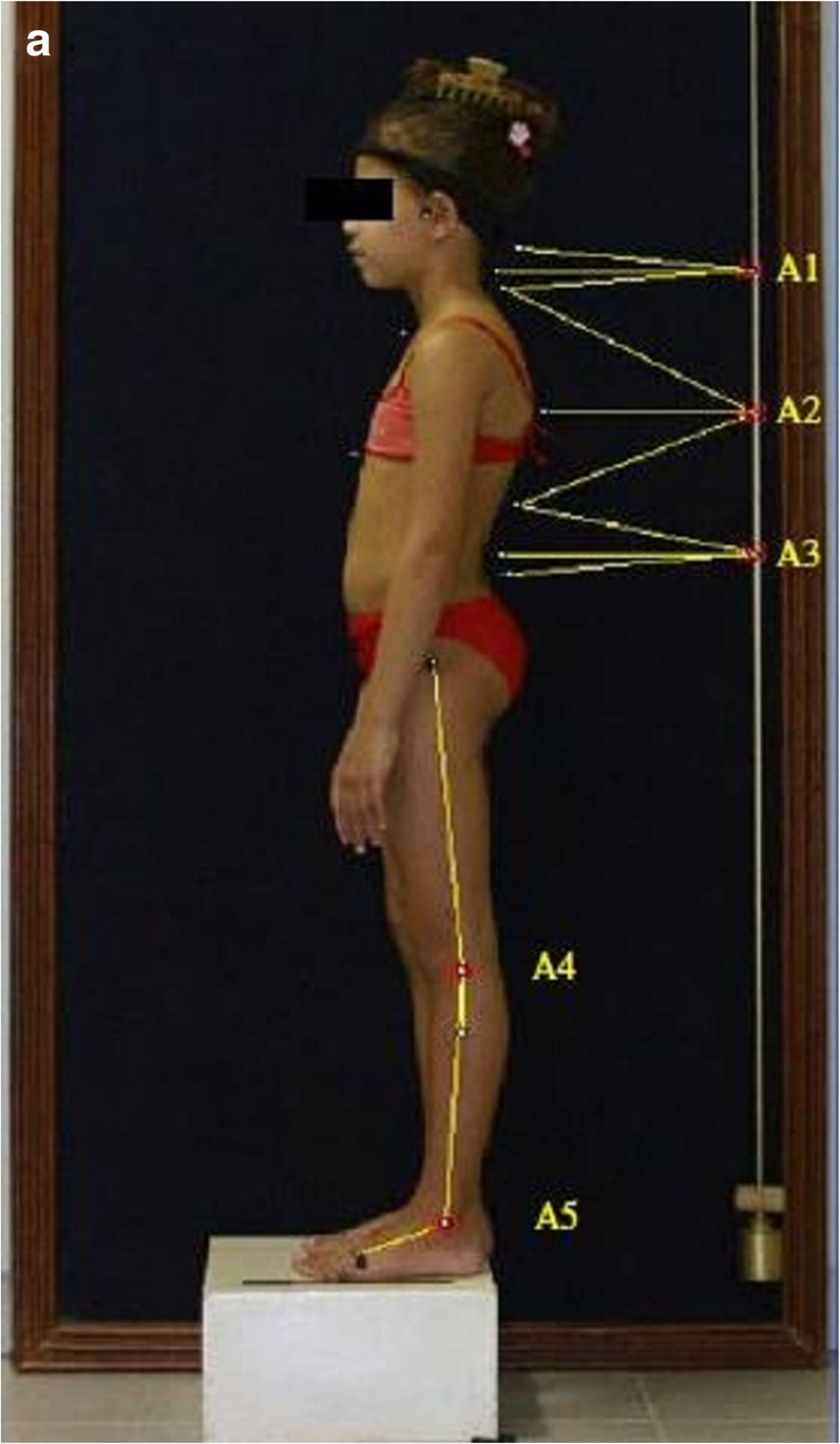
Photographic assessment as proposed by Canhadas et al. in 2009 ([74], reprinted by permission)

Canhadas et al. [74] proposed the following anatomical points to be considered: external orbicularis, commissura labiorum, acromioclavicular joint, sternoclavicular joint, ear lobe, antero-superior iliac spines, postero-superior and postero-inferior iliac spines, inferior angles of the scapula, olecranon central region, and popliteal line. In addition, the following angles were evaluated: bilateral foot inclination, forward inclination of the fibula, knee angle, cervical lordosis, thoracic kyphosis, lumbar lordosis, knee flexor, tibiotarsal angle, forward head position, and sternal angles (Fig. 32).

**Fig. 33:**
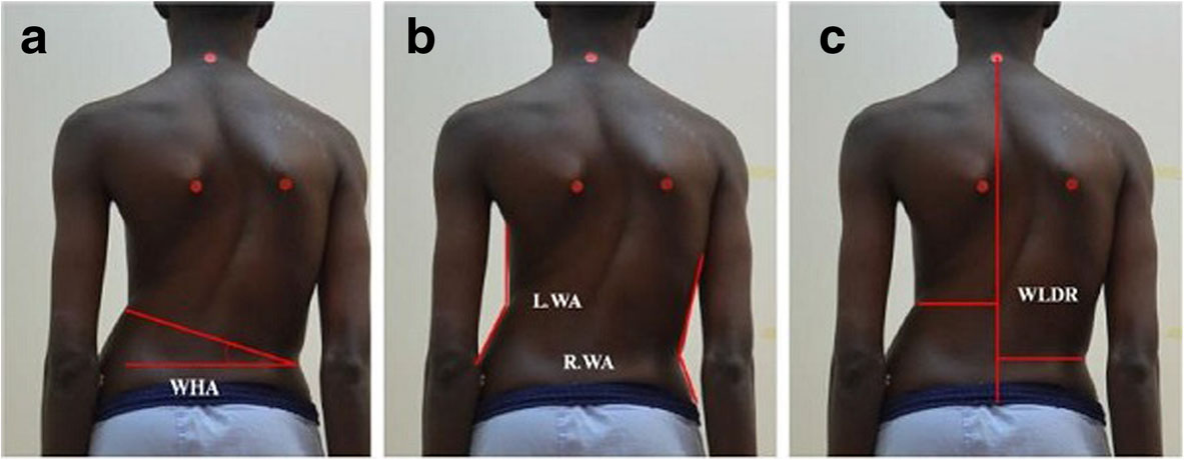
**a**–**c** Photographic assessment as proposed by Matamalas et al. in 2016 ([75], reprinted by permission)

Matamalas et al. [75] reported the following posterior parameters: waist height angle, waist angle, and waistline distance ratio (Fig. 33).

**Fig. 34:**
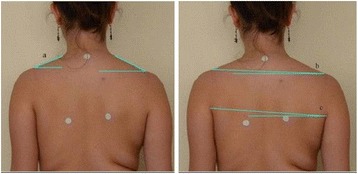
Photographic assessment as proposed by Matamalas et al. in 2014 ([76], reprinted by permission)

Matamalas et al. [76] published the following anterior and posterior parameters: trapezium angle, shoulder height angle, and axilla height angle (Fig. 34).

### Current opinions on digital photography technique

Digital photography completed with analyzing software can be viewed as digital photogrammetry and can be found in several areas of life and technology: architecture, psychology, medicine, rehabilitation, and other fields [77–80]. For the purpose of posture assessment, this technique is easy to access and cost-effective [81, 82]. The technique provides measurement of body angles or distances, which allows for a quantitative posture assessment. Remaining non-invasive, digital photography is becoming an increasingly popular tool for assessing the musculoskeletal system, including the sagittal and coronal curvatures of the spine, in both clinical practice and research [83, 84]. In recent years, the photographic technique has been used to assess the posture of healthy and unhealthy children and adults [69, 74, 85]. Digital photography was applied to assess body posture of children carrying heavy backpacks [86], to evaluate the quality of posture while standing [87, 88] and siting [89], or for quantifying the foot shape [90]. Several studies described usefulness of the photographic technique to assess patients with idiopathic scoliosis [75, 91–94].

### Technical procedures of posture photogrammetry

#### Camera resolution

Different resolutions of digital cameras were used in the previous studies, ranging from 2.0 megapixels (Mpx) [73], 4.1 Mpx [95, 96], through 5.1 Mpx [84], 6.0 Mpx [81], 6.3 Mpx [97] to 7.2 Mpx [98]. For this study, CANON POWER SHOT A590 IS, 1/2.5 CCD matrix, 8.3 megapixels, 35–140-mm lens (Canon Incorporation, Tokyo, Japan) was used. The resolution of 1600 × 1200 [2 Mpx] provided sufficient photo quality [99].

#### Camera position—distance and height

In previous photographic studies, the distance between the camera and the object was reported to be 173 cm [97, 100], 300 cm [73, 81, 95, 101], or 400 cm [55]. The camera was positioned at the height of 70, 127, 80 or 90 cm [81], while other authors set the camera by centering the lens at half of the child’s height [81, 95, 98]. In our previous experiments, the camera was placed on a stabile tripod at the height of 90 cm and the distance of 300 cm. These settings were previously suggested for children aged 7–10 [81, 98]. Such a combination of distance and height enabled covering the whole silhouette without moving the camera [12].

#### Child positioning

Some authors proposed to practice the photographic examination of the standing child wearing casual clothes, sportswear (shorts and a T-shirt) [87], just shorts [64], or the swimsuit [102]. Unfortunately, the clothes may slightly distort the body contour. Producing and registering images of undressed children seems to be a potential challenge for posture photogrammetry. Nowadays, it involves both the imperative to adopt procedures respecting individual sensitivity and the protection of image processing and storing. Yet, here we are, proposing the evaluation of the child body posture without any T-shirt, thigh or socks, wearing only underwear and bra [103], which is not commonly accepted in our society (with individual cases of parents refusal noted). However, the local cultural background should be considered. Longer hair of the person examined should be tied or curled with a clip so as not to cover the external auditory meatus or neck contour.

#### Lower limb positioning—the feet

In previous studies, some authors proposed to set the feet at the 30-degree external rotation in drawn triangles [97] or freely within the defined field lines [84]. In our observation, the 30-degree external rotation of the feet may undesirably impact the position of other parts of the body, especially the ankle joint in relation to the vertical projection of the quadrangle support. We decided to position the feet over the longitudinal and crosswise lines marked on the ground so that the lateral malleoli were situated over the center of crosswise line, and the feet were parallel to the longitudinal line and hip-width apart. We found such setting to be the most neutral feet position which does not interfere with the spontaneous posture [63]. It has also the advantage of being suitable for assessing the tibio-calcaneal angle. In our experiments, most children needed assistance to place the feet correctly.

#### Lower limbs positioning—the knees

The position of the knee joint and the symmetric lower limbs loading are also objects for standardization as some children tend to stand for the photographic evaluation having one lower limb more loaded or one knee more visibly bended. Such a position would influence the whole body posture, especially the trunk. We recommend positioning the child with equally loaded feet, in neutral setting of the knees, without flexion or hyper-extension.

#### Upper limb positioning—the elbows

Most authors suggest using the position with upper limbs hanging loosely [87, 96, 98, 102, 104, 105] in order not to influence the trunk [106]. The problem of the upper limb positioning is well-studied in the case of lateral spine radiography and different solutions are proposed to avoid the spine being obscured by the upper limbs [106–108]. Moreover, in the course of the standardization studies, we observed that the relaxed upper limbs sometimes covered the lumbar lordosis contour and greater trochanter. Similar observations have been made by other authors who suggested carrying out photographic sagittal evaluation with the elbow joints bent at 90° [73, 87, 109]. Finally, we recommend setting the upper limbs slightly flexed at about 10°–20° at the gleno-humeral joints and at about 20°–30° at the elbow joints. The movement of the upper limb flexion in the gleno-humeral and the elbow joints is performed slowly to avoid any involuntary trunk movement towards trunk hyperextension [87], which is the way to increasing lower thoracic spine [110] or even creating a pathological lordosis in this region [111]. During this movement, the child is watched, and if any accompanying trunk movement happens, the child is asked to repeat the upper limb movement. In some cases, passive positioning of the upper limbs is needed. In addition, we observed that during the upper limb movement, some children performed elevation or protraction of the shoulders which covered the neck contour and the upper thoracic spine contour. Therefore, during the positioning of the upper limbs, we make sure that the shoulder girdle stays down. It is important to note that the presence of shoulder protraction in loosely hanging upper limbs is common in the population of children aged 7–10 [8].

#### Head position and gaze direction

During the standardization of the photographic technique, we checked the effect of the head position and the gaze direction on postural parameters. Our preliminary studies have shown that the head position affects the angular size of thoracic kyphosis and lumbar lordosis. Initially, we were planning to ask the child to look at a specific point marked in front of her/him as proposed in the literature [87]. Then, we noted this created an additional problem because of differences in children’s height, which is why we proceeded with the “look ahead” command. Nevertheless, we noticed that some children, even when looking ahead, maintained voluntary lowered head position with the flexion of the cervical spine. Therefore, in order to achieve standardized conditions, each child was instructed to keep the eyes open and to direct the gaze at the eye level the moment it receives the “look ahead” command [71, 109, 112, 113]. Consequently, if an inappropriate voluntary head position was observed, we explained to the child once again how the head should be set. In rare situations, we helped the child by modifying the head position in a gentle way, trying not to trigger any artificially corrected position. In our practice, we also found it useful to ask children not to smile or laugh while taking the photos as it could affect postural parameters [112].

### Digital photography technique for body posture evaluation and documentation

During this study, the postural photogrammetry revealed a simple and quick procedure. One can possibly perform photographic measurements with the use of a simple digital camera or a mobile camera in consideration of the standardized conditions for photographic evaluation. The tripod revealed a helpful device to stabilize the camera and control its position. The time needed for preparing the child for examination together with the time for taking photographic exposures in two sagittal projections was ca. 5 min, whereas the time required for calculation of five standardized sagittal parameters was ca. 3 min. This study confirmed the usefulness of photographic method for body posture documentation and evaluation. Digital photography technique can be used in research on the development and variability of posture in children. The developed procedure allows for the accurate and uniform filling of photographic documentation by physiotherapists and to obtain good quality research which is in line with the EBM rules. Due to its non-invasiveness, the technique can be promoted in scientific and clinical research. Parents’ concerns regarding the use of radiography are avoided. The low cost of producing and archiving digital photos has a beneficial effect on technology. There is no need to acquire expensive, specialized equipment or software. Digital photogrammetry screening can be significant for the budget savings of individual units which organize screening (e.g., the local government), which is often crucial in financing various types of research projects. Specific numerical values of the normal range for quantitative and validated parameters are presented in this paper. According to van Maanem et al., the simplicity of assessing the posture on the photos is at the core of this technique—it is objective, easy to use, and of low cost [114]. For Cobb et al. [90], the digital photography for a two-dimensional assessment of the body shape is a valuable method for recording the body posture and calculating quantitative parameters in everyday clinical practice. Fortin et al. [109] claim that digital photography technique can be used for scientific assessment provided that the procedures in question are taken into account. Galera et al. mention that the current studies present new diagnostic possibilities of digital photography, which is a common procedure for two-dimensional evaluation of body posture [71]. Digital photography has some limitations. The major limitation of the technique is the two-dimensional body posture assessment, as it is not impossible to measure trunk rotation. The method may not be suitable for children under 7 years of age.

## Conclusions

In summary, although both the surface topography and the radiological evaluation cannot be replaced with digital photography—the former for the 3D imaging, the latter for skeletal imaging—this technique offers a new additive value to human posture imaging. The development of digital photography technique allows for its regular use in the assessment of body posture. The method of child preparation and positioning described above allows us to avoid incidentally modified posture. The registration of images is simple, quick, harmless and cost-effective. The semi-automatic image analysis has been developed. The choice of postural parameters was based on previous publications and on personal experience and can be modified. The photographic method of body posture assessment developed during this study is characteristic of high reliability of measurements. The five developed and calculated photographic parameters (sacral slope, thoracic kyphosis, lumbar lordosis, chest inclination, and head protraction) describe the child body posture in the sagittal plane and demonstrate good repeatability and reproducibility, which may become a standard for body posture evaluation in children. Performing such a large series of measurements in children resulted in the preparation of normal values and percentile charts for age and sex, making it possible for us to employ the photographic parameters possible in the diagnosis of child posture pathology as well as to monitor the effects of corrective therapy.

## Additional files

Additional file 1: Appendix 1. Coronal and sagittal photographic parameters. (PDF 268 kb)

Additional file 2: Appendix 2. Normative values of sagittal photographic parameters for children aged 7–10 based on the tables and percentile charts for sex and age. (PDF 1000 kb)

## Abbreviations

AA: Acromion-ankle angle
ASIS: Anterior superior iliac spine
ATSI: Anterior Trunk Symmetry Index
C7: Seventh cervical vertebra
CI: Chest inclination angle
EA: Ear-ankle angle
EBM: Evidence-based medicine
FAI-A: Frontal Asymmetry Index at axilla level
FAI-C7: Frontal Asymmetry Index at C7 level
FAI-SN: Frontal Asymmetry Index at sternal notch level
FAI-T: Frontal Asymmetry Index at trunk level
HDI-A: Height Difference Index for axillas
HDI-S: Height Difference Index for shoulders
HDI-T: Height Difference Index for trunk waistline
HP: Head protraction angle
ICC: Intraclass correlation coefficient
IS: Idiopathic scoliosis
L1: First lumbar vertebra
L5: Fifth lumbar vertebra
LL: Lumbar lordosis angle
POTSI: Posterior Trunk Symmetry Index
PSIS: Posterior superior iliac spine
S1: First sacral vertebra
SEM: Standard error for single measurement
SPT: Sagittal pelvic tilt
SS: Sacral slope angle
T12: Twelfth thoracic vertebra
TA: Trochanter-ankle angle
TCA: Tibio-calcaneal angle
TFA: Tibio-femoral angle
TK: Thoracic kyphosis angle
