# Supplementary material for: Two-dimensional digital photography for child body posture evaluation: standardized technique, reliable parameters and normative data for age 7-10 years
Source: Scoliosis Spinal Disord. 2017 Dec 19;12:38. doi: 10.1186/s13013-017-0146-7 (PMC5738151; doi:10.1186/s13013-017-0146-7)
Supplement: Supplementary file 1 — Appendix 1. Coronal and sagittal photographic parameters. (PDF 268 kb) [file 13013_2017_146_MOESM1_ESM.pdf]

**Appendix 1**

Coronal and sagittal photographic parameters

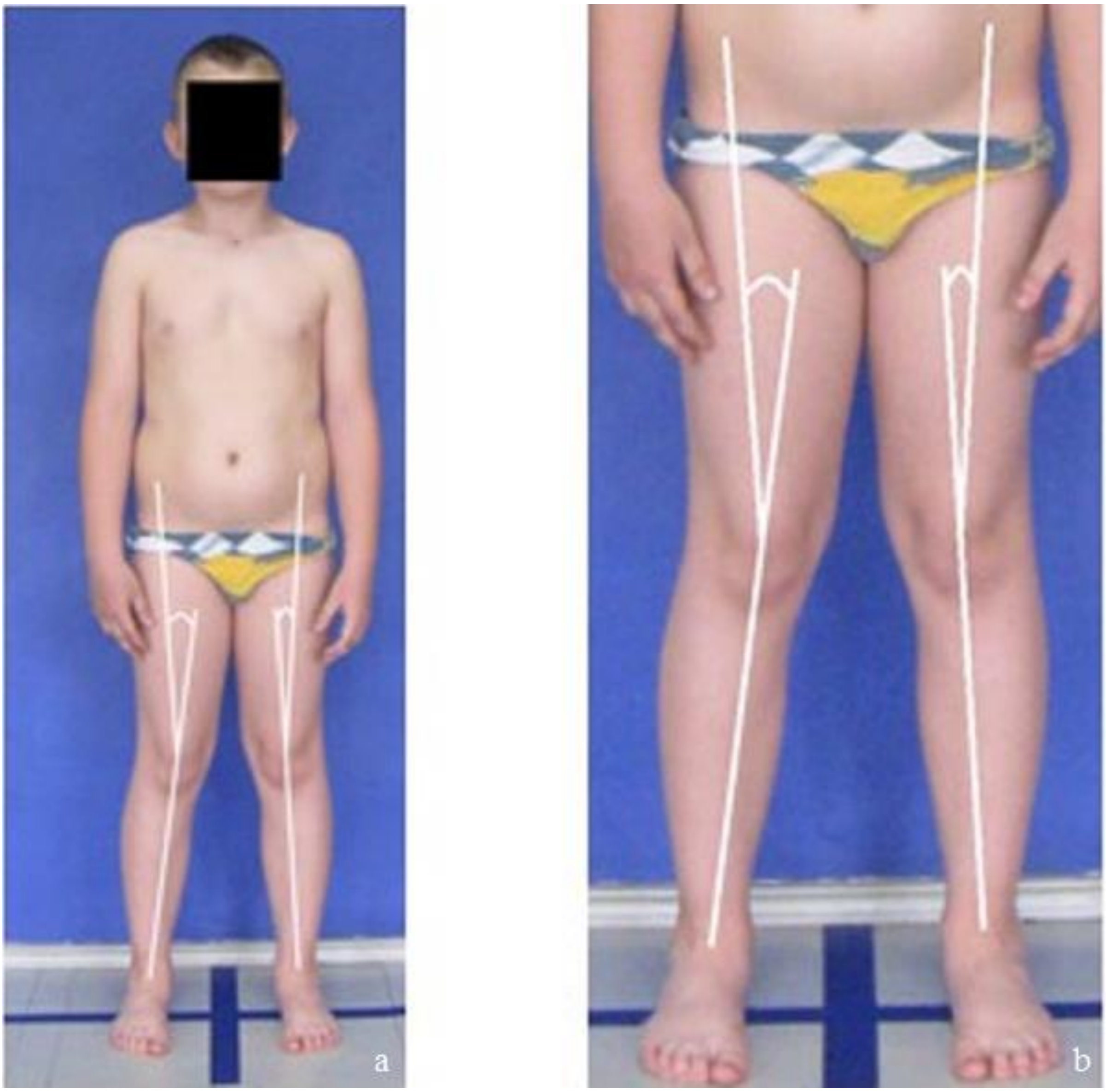


Figure 8A. Diagram illustrating the measurements of Tibio-Femoral Angle (TFA). a actual size; b enlarged size.

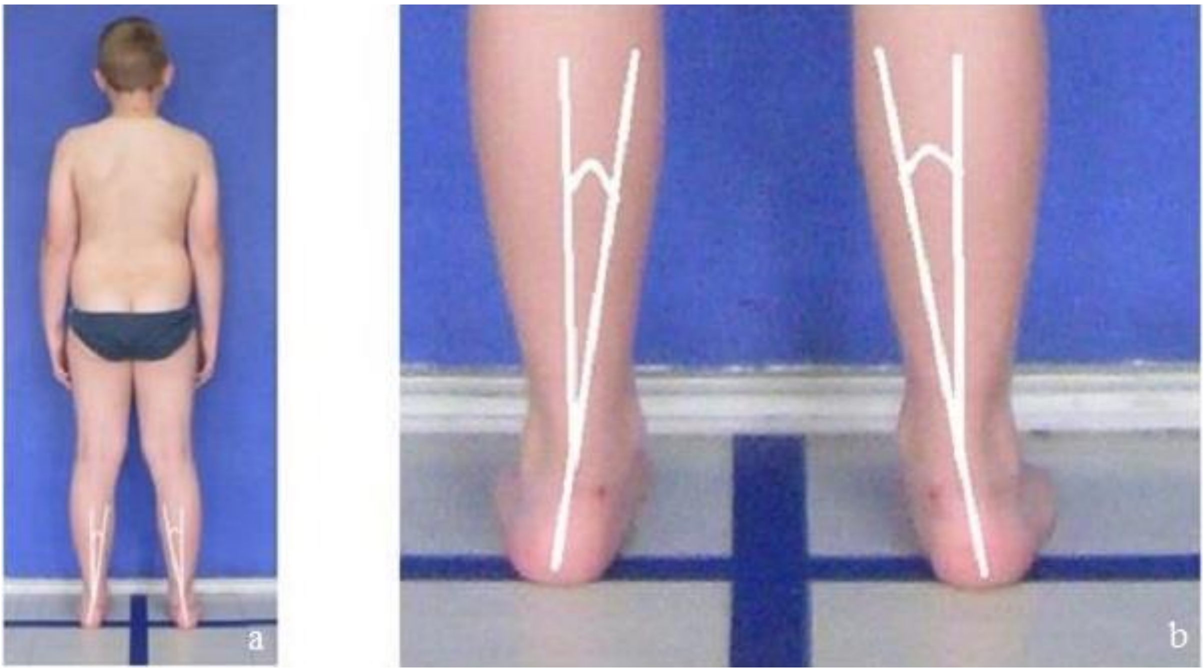


Figure 8B. Diagram illustrating the measurements of Tibio-Calcaneal Angle (TCA). a actual size; b enlarged size.

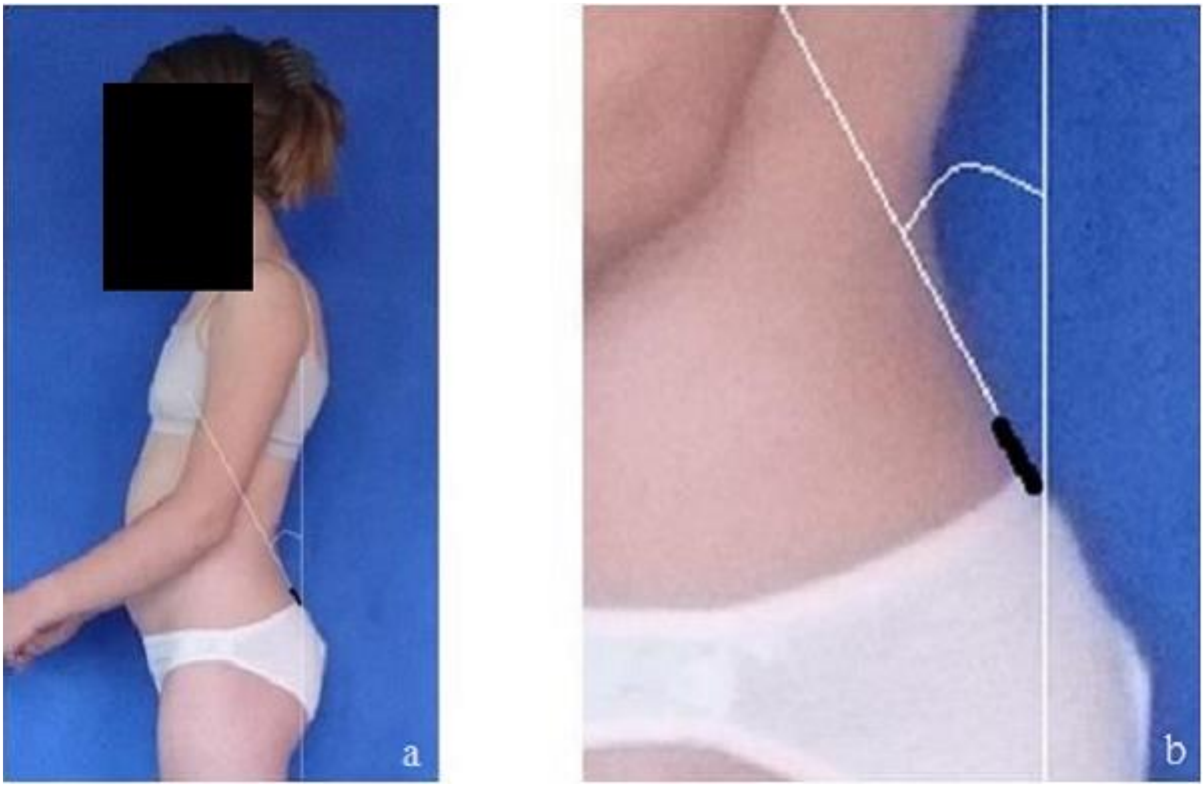


Figure 9A. Diagram illustrating the measurements of Sacral Slope angle (SS). a actual size; b enlarged size.

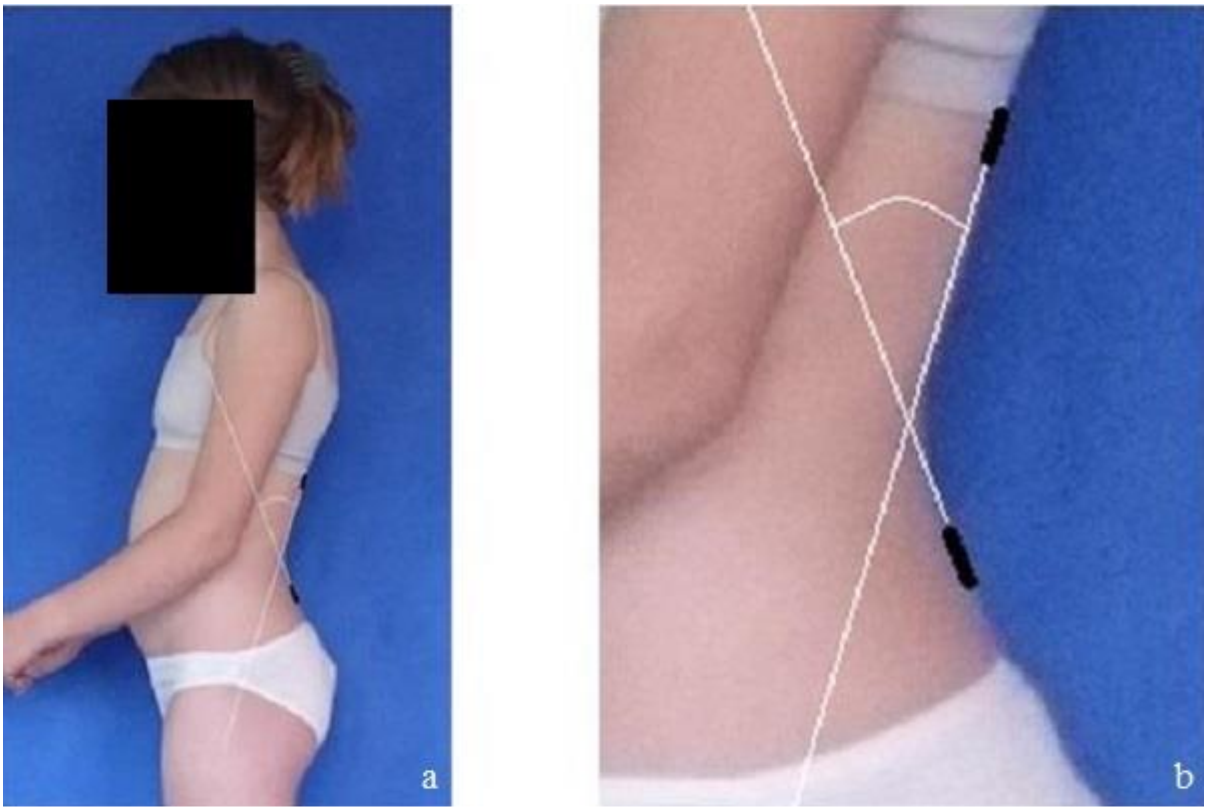


Figure 9B. Diagram illustrating the measurements of Lumbar Lordosis angle (LL). a actual size; b enlarged size.

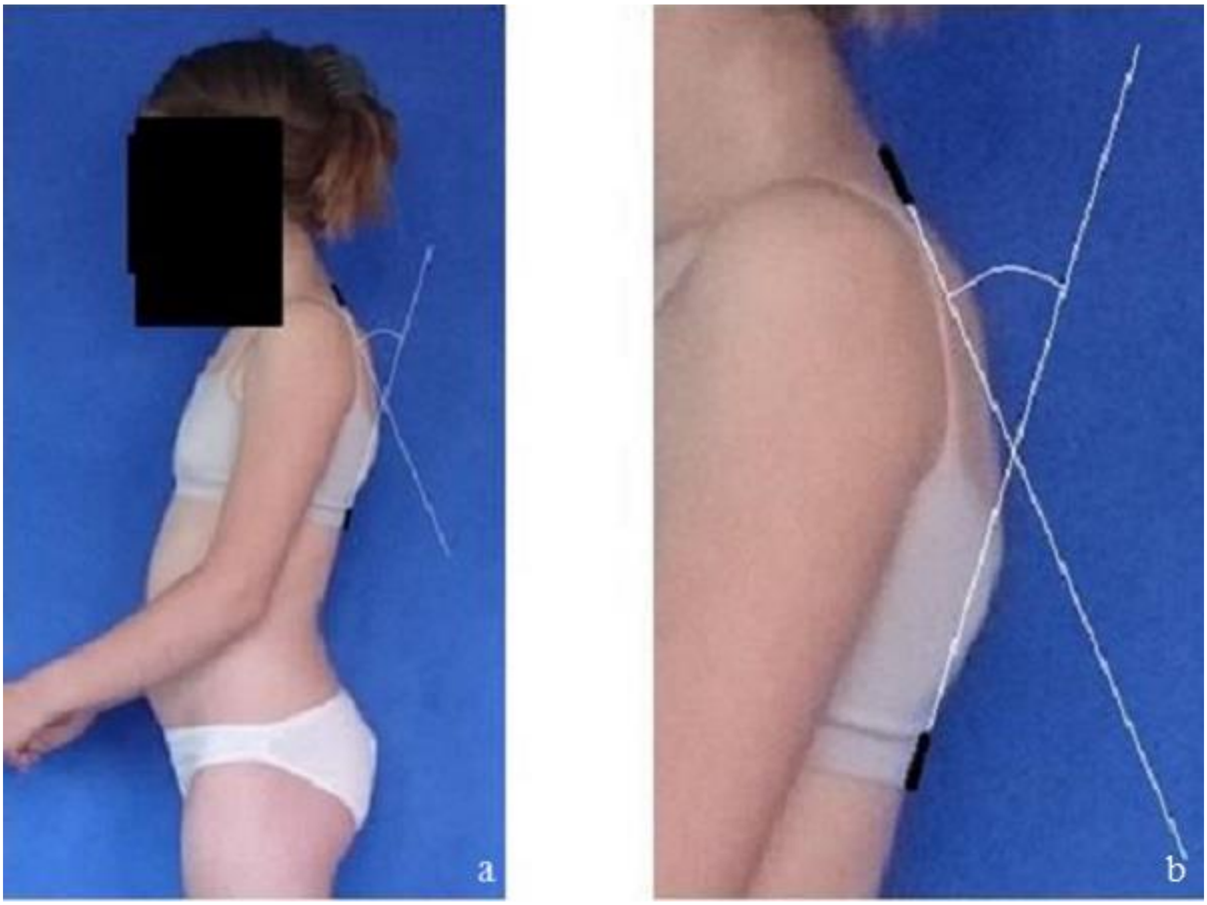

Figure 9C. Diagram illustrating the measurements of Thoracic Kyphosis angle (TK). a actual size; b enlarged size.

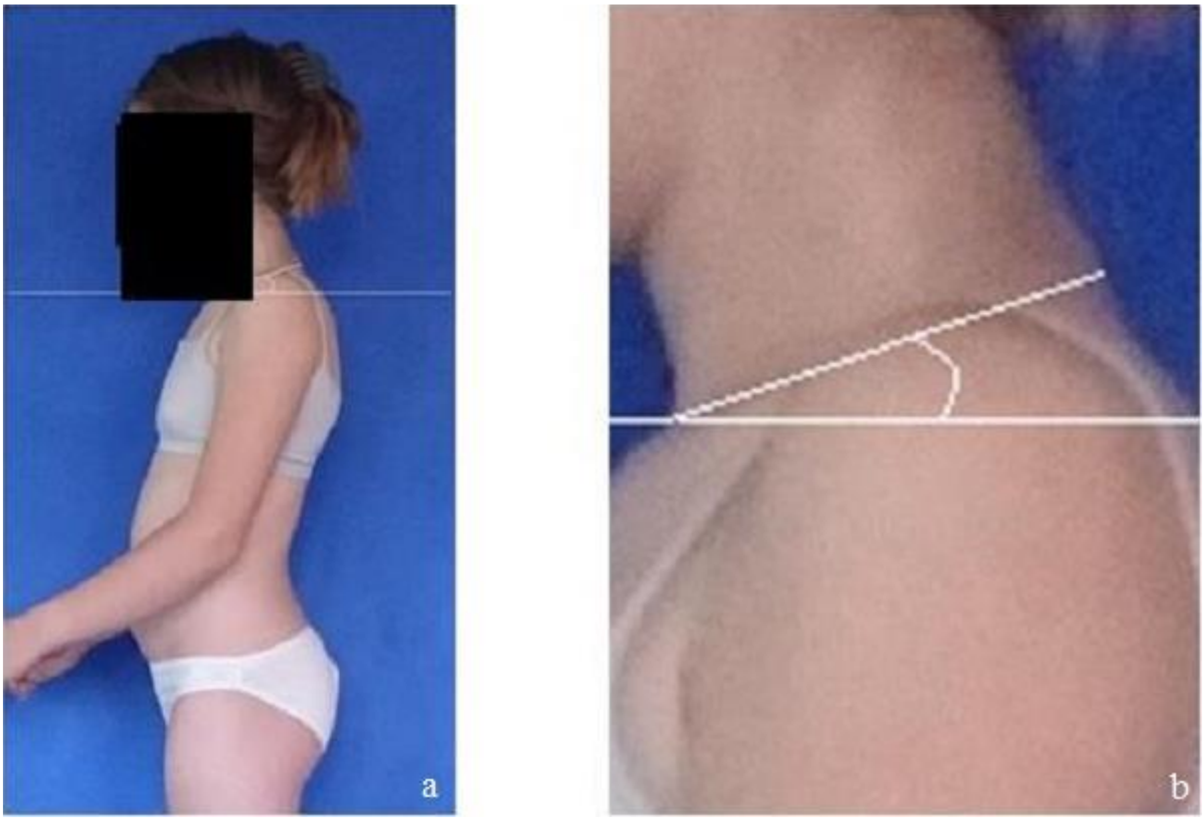


Figure 9D. Diagram illustrating the measurements of Chest Inclination angle (CI). a actual size; b enlarged size.

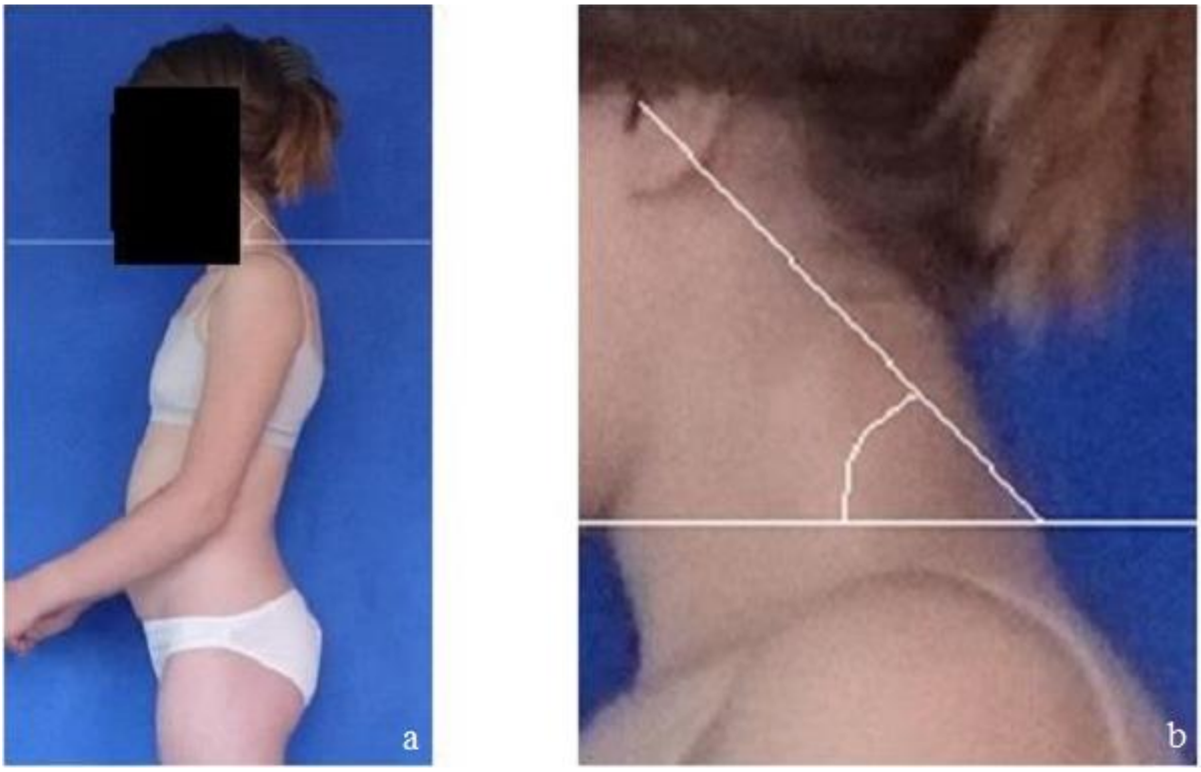

Figure 9E. Diagram illustrating the measurements of Head Protraction angle (HP).  a actual size; b enlarged size.

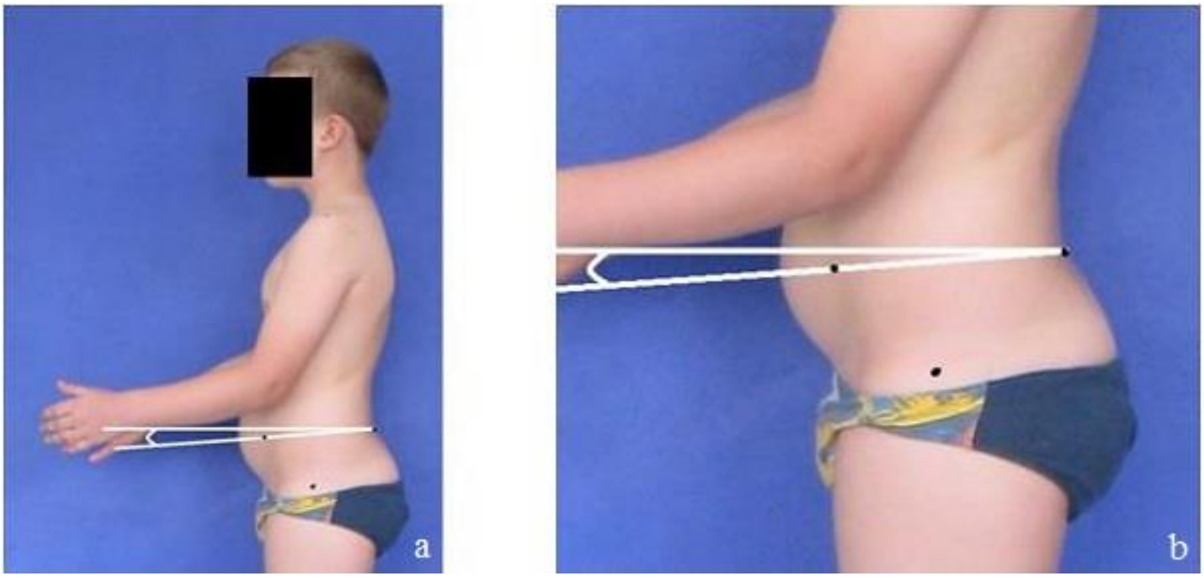


Figure 10A. Diagram illustrating the measurements of Sagittal Pelvic Tilt (SPT). a actual size; b enlarged size.

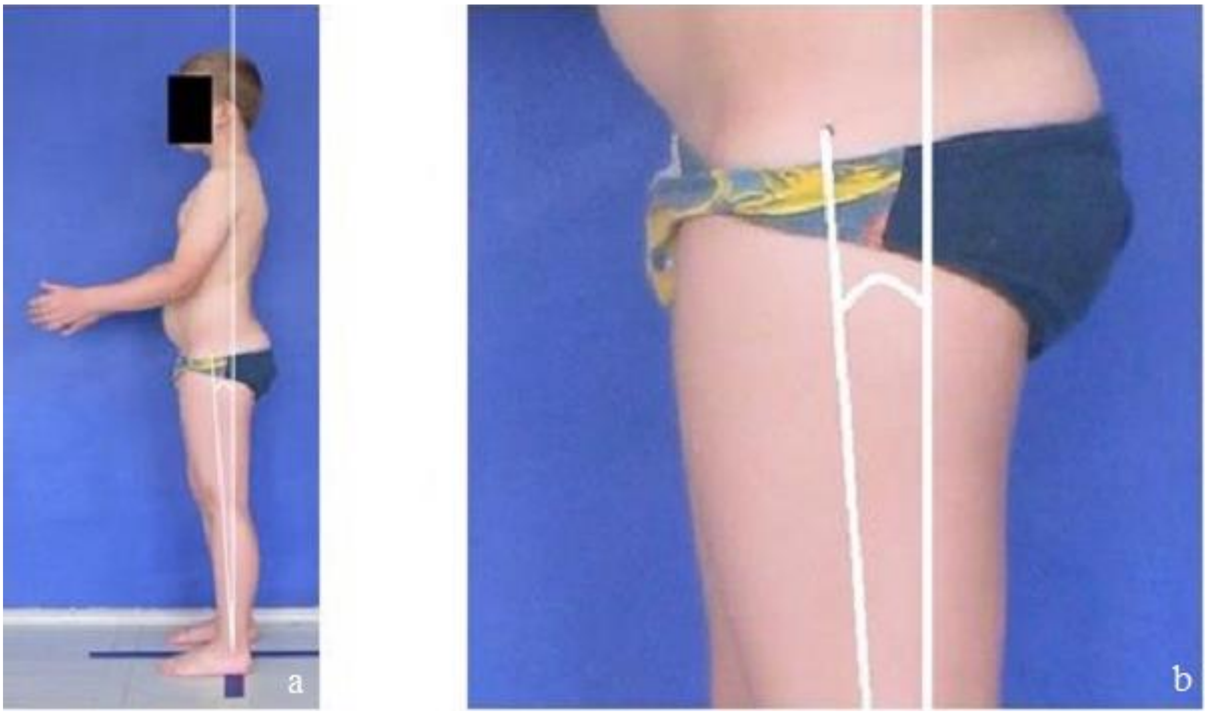


Figure 10B. Diagram illustrating the measurements of Trochanter-Ankle angle (TA). a actual size; b enlarged size.

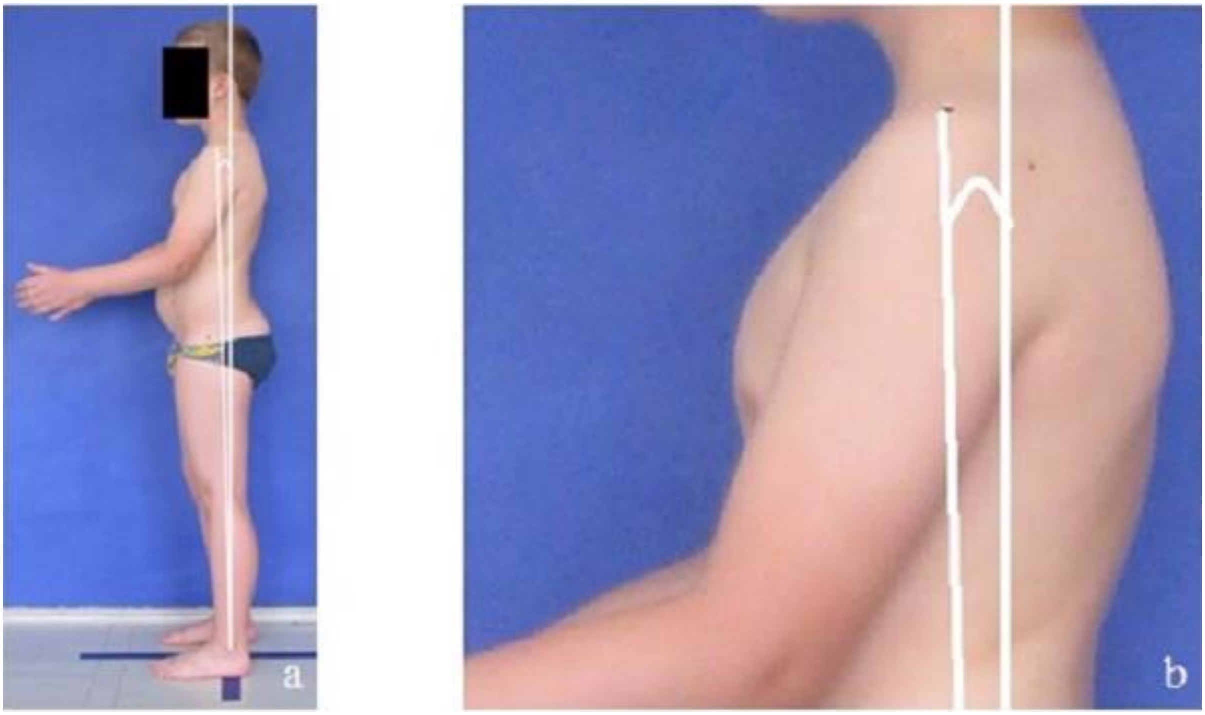


Figure 10C. Diagram illustrating the measurements of Acromion-Ankle angle (AA). a actual size; b enlarged size.

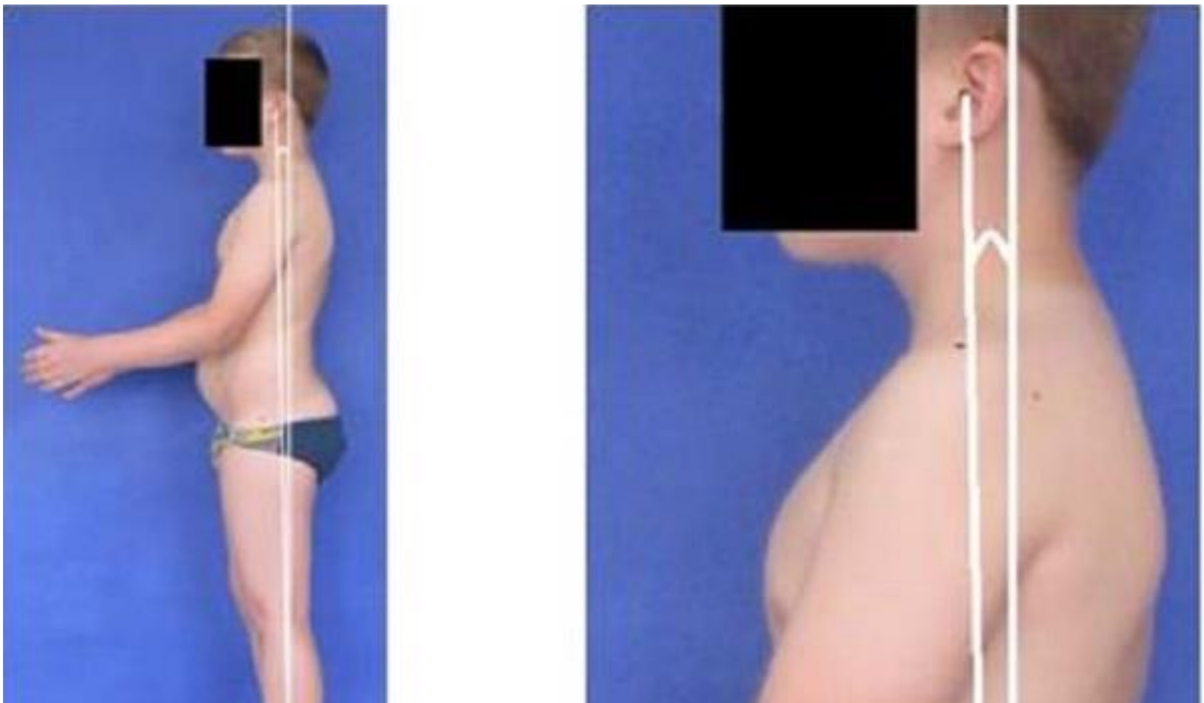


Figure 10D. Diagram illustrating the measurements of Ear-Ankle angle (EA). a actual size; b enlarged size.
