# Supplementary material for: Two-dimensional digital photography for child body posture evaluation: standardized technique, reliable parameters and normative data for age 7-10 years
Source: Scoliosis Spinal Disord. 2017 Dec 19;12:38. doi: 10.1186/s13013-017-0146-7 (PMC5738151; doi:10.1186/s13013-017-0146-7)
Supplement: Supplementary file 2 — Appendix 2. Normative values of sagittal photographic parameters for children aged 7–10 based on the tables and percentile charts for sex and age. (PDF 1000 kb) [file 13013_2017_146_MOESM2_ESM.pdf]

Appendix 2

Appendix 2A. Normative values of sagittal photographic parameters for children aged 7-10 years based on the tables for sex and age.

Table 4. Numerical values for 7-years old girls (N=1083)

**Percentile**	**SS**	**LL**	**TK**	**CI**	**HP**
97	44	52	62	41	71
90	39	45	55	36	66
75	33	37	48	32	63
**50**	**27**	**29**	**42**	**27**	**58**
25	22	23	35	22	54
10	17	18	28	18	49
3	12	13	23	14	45

Table 5. Numerical values for 8-years old girls (N=915)

**Percentile**	**SS**	**LL**	**TK**	**CI**	**HP**
97	45	52	62	41	69
90	38	44	55	36	66
75	33	37	48	32	62
**50**	**28**	**30**	**42**	**27**	**58**
25	23	24	35	22	54
10	18	20	29	18	49
3	14	14	23	14	45

Table 6. Numerical values for 9-years old girls (N=936)

Percentile	SS	LL	TK	CI	HP
97	44	51	64	39	69
90	38	44	57	36	65
75	34	37	51	32	63
**50**	**28**	**31**	**43**	**27**	**58**
25	23	24	36	23	55
10	18	20	31	19	51
3	13	14	24	15	47

Table 7. Numerical values for 10-years old girls (N=870)

Percentile	SS	LL	TK	CI	HP
97	43	52	64	41	69
90	37	45	57	36	66
75	32	38	51	32	63
**50**	**27**	**32**	**43**	**27**	**58**
25	22	25	36	23	54
10	18	21	31	19	50
3	13	16	25	13	45

Table 8. Numerical values for 7-years old boys (N=1167)

Percentile	SS	LL	TK	CI	HP
97	44	48	44	39	72
90	39	40	39	36	68
75	33	32	33	32	64
**50**	**27**	**25**	**27**	**27**	**59**
25	22	20	22	22	55
10	16	15	16	18	52
3	11	12	11	13	46

Table 9. Numerical values for 8-years old boys (N=990)

Percentile	SS	LL	TK	CI	HP
97	44	48	63	39	72
90	39	41	55	35	68
75	33	34	50	32	64
**50**	**26**	**27**	**43**	**26**	**60**
25	21	21	36	22	55
10	17	17	32	19	52
3	11	12	24	13	46

Table 10. Numerical values for 9-years old boys (N=929)

Percentile	SS	LL	TK	CI	HP
97	43	46	64	41	72
90	38	39	58	36	68
75	33	33	52	32	64
**50**	**26**	**27**	**44**	**27**	**60**
25	22	22	37	23	55
10	17	17	32	18	52
3	13	13	24	14	47

Table 11. Numerical values for 10-years old boys (N=892)

Percentile	SS	LL	TK	CI	HP
97	44	47	66	41	72
90	37	38	58	36	67
75	32	33	52	33	64
**50**	**27**	**26**	**44**	**27**	**60**
25	22	22	37	23	55
10	16	17	31	17	52
3	12	12	25	15	47

Appendix 2B. Normative values of sagittal photographic parameters for children aged 7-10 years based on the percentile charts for sex and age.

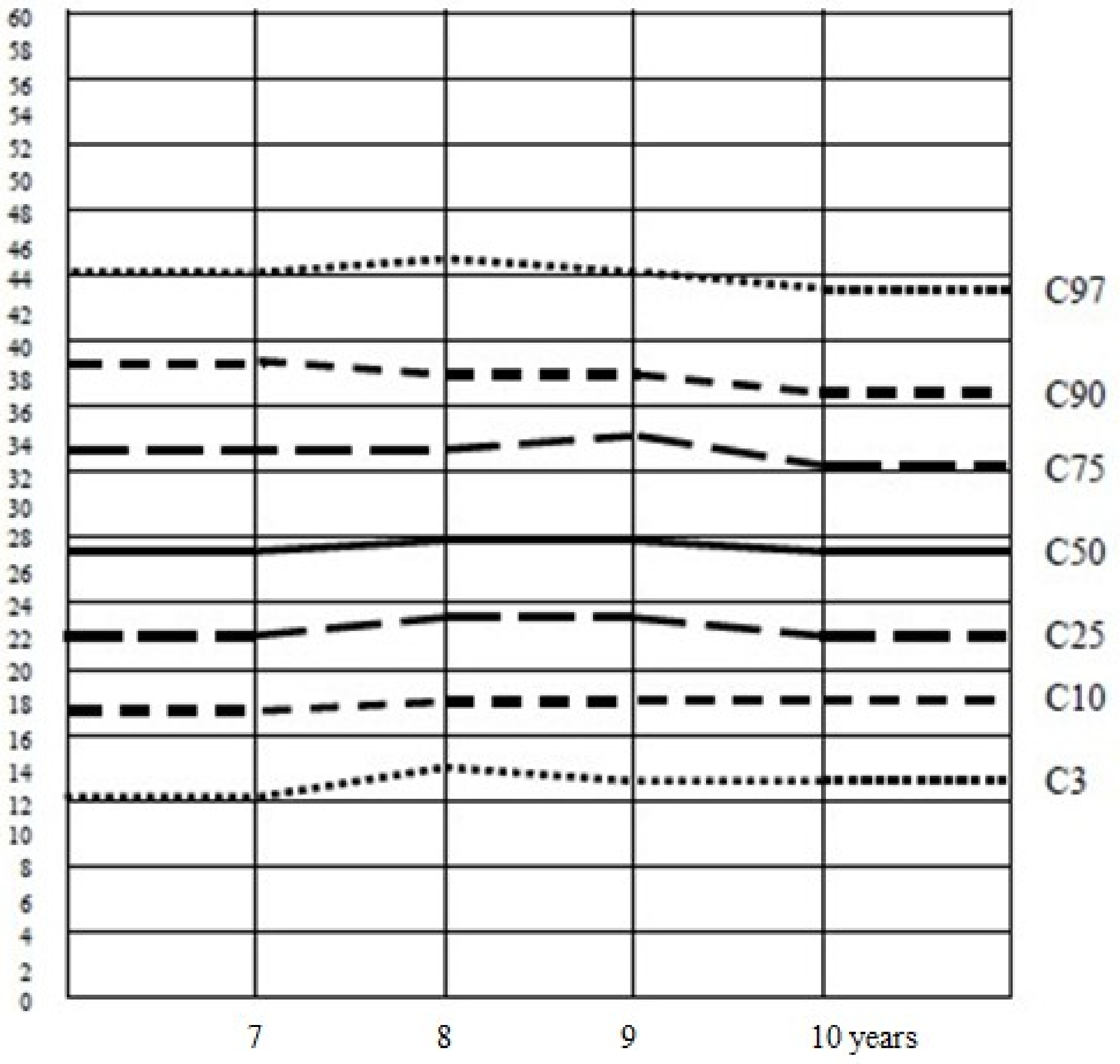

Figure 1. Sacral Slope Angle for girls (SS)

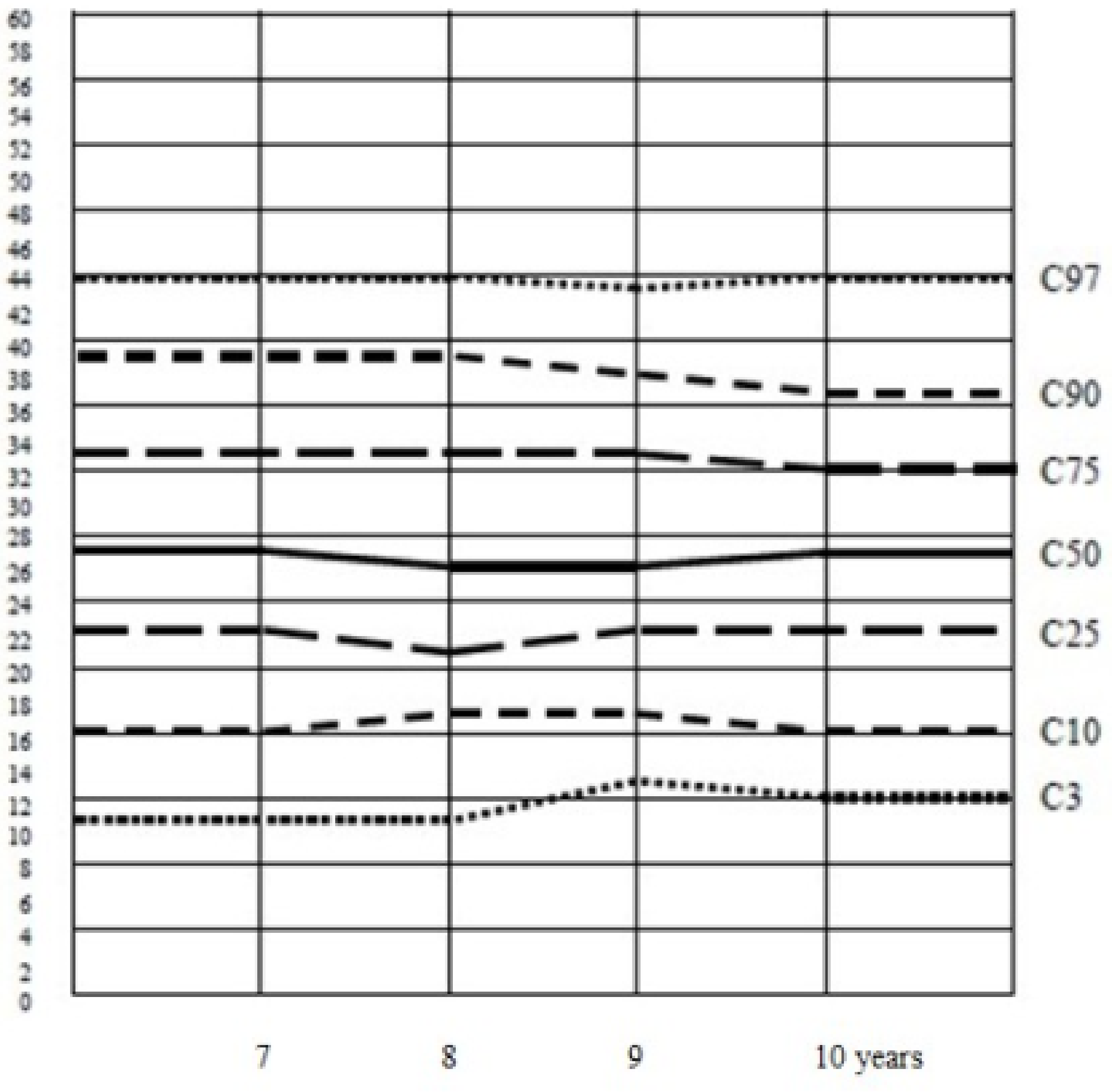

Figure 2. Sacral Slope Angle for boys (SS)

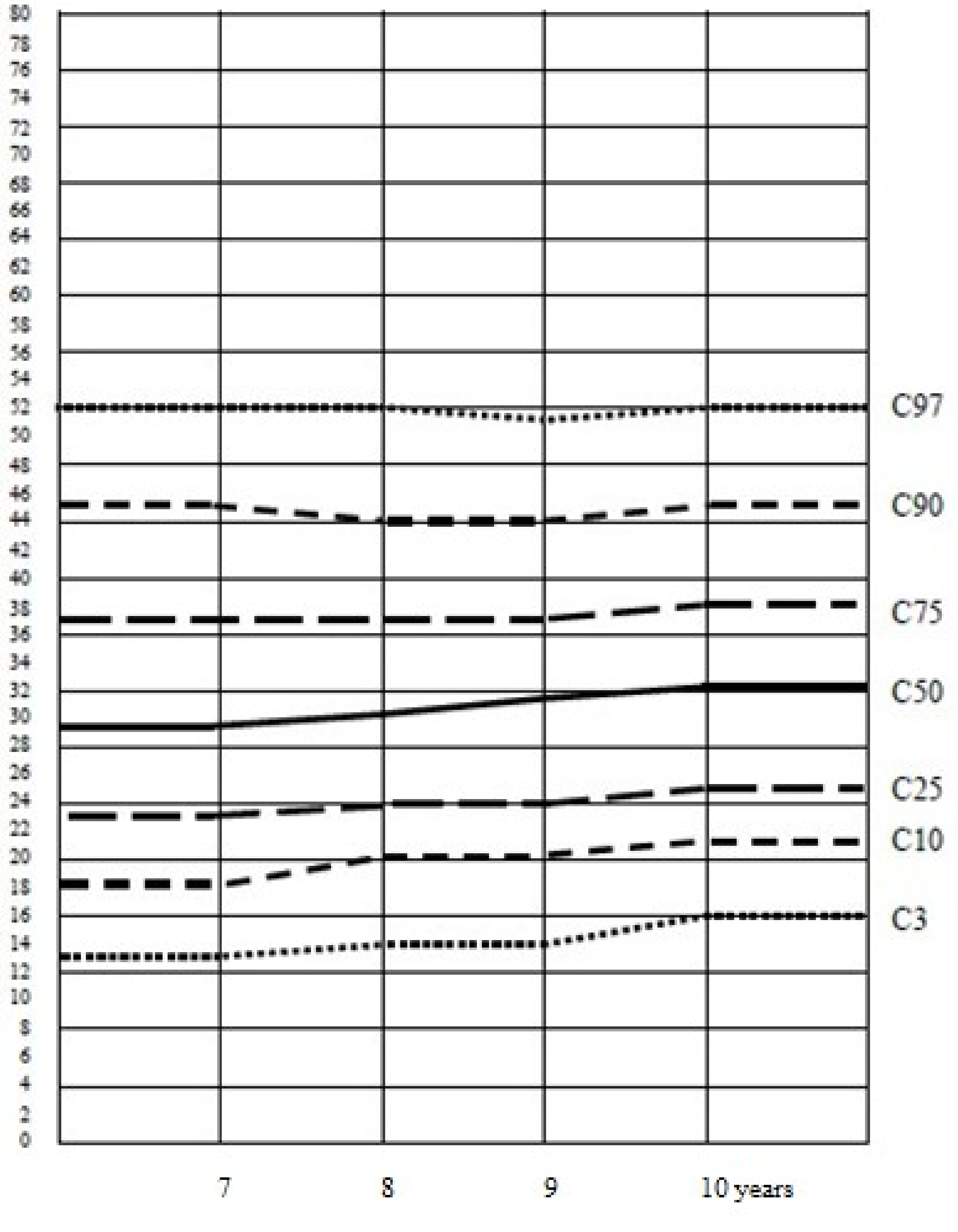

Figure 3. Lumbar Lordosis Angle for girls (LL)

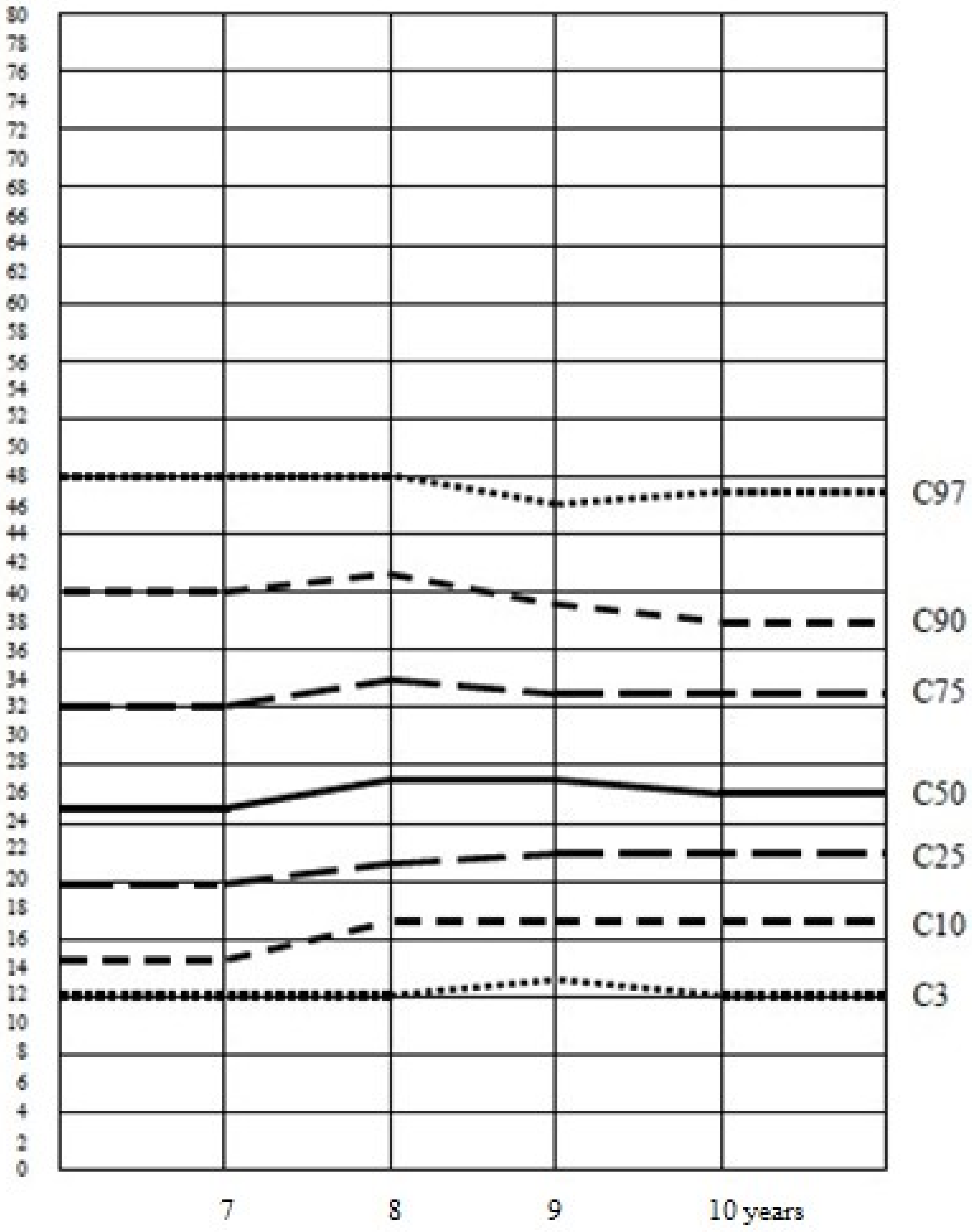

Figure 4. Lumbar Lordosis Angle for boys (LL)

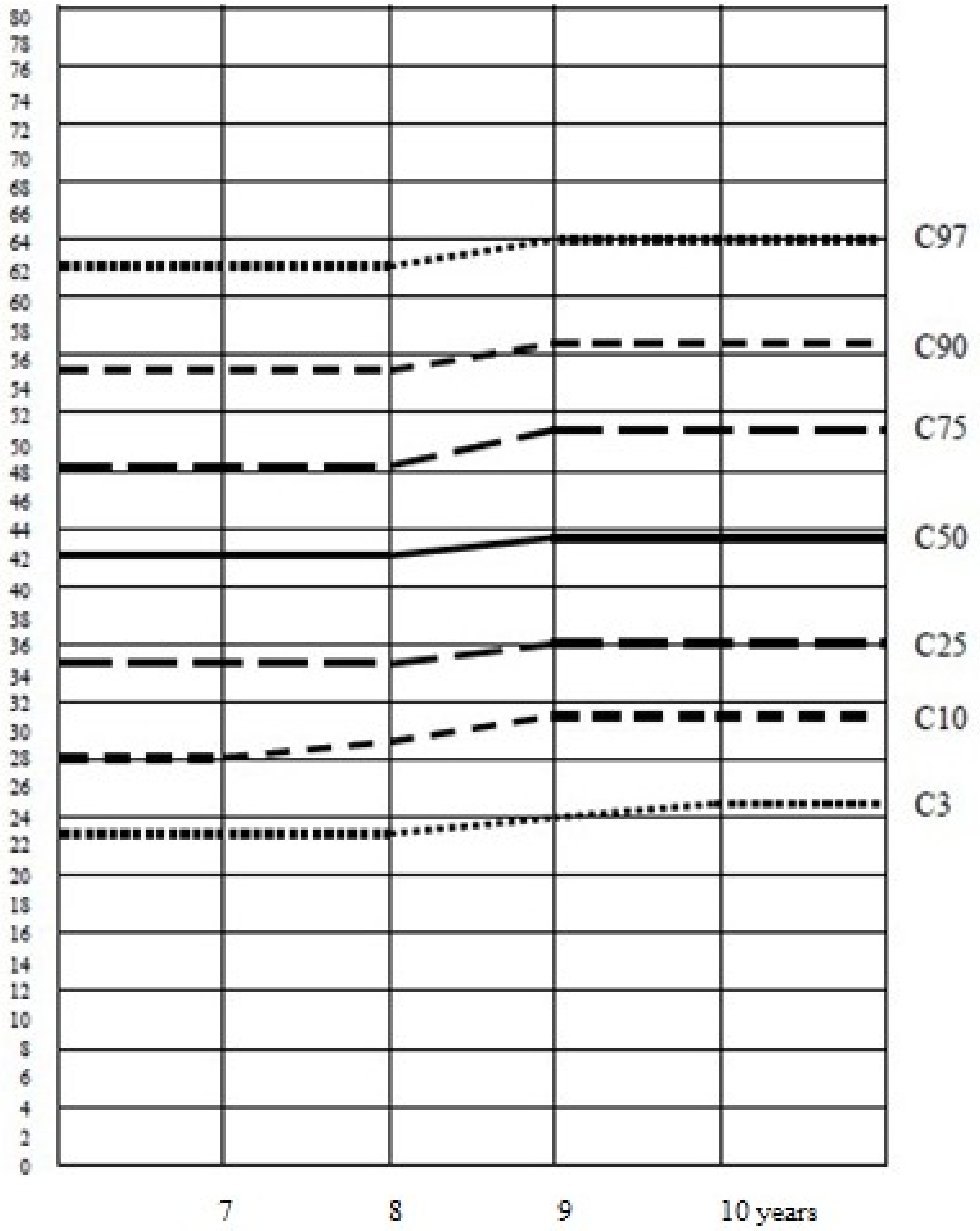

Figure 5. Thoracic Kyphosis Angle for girls (TK)

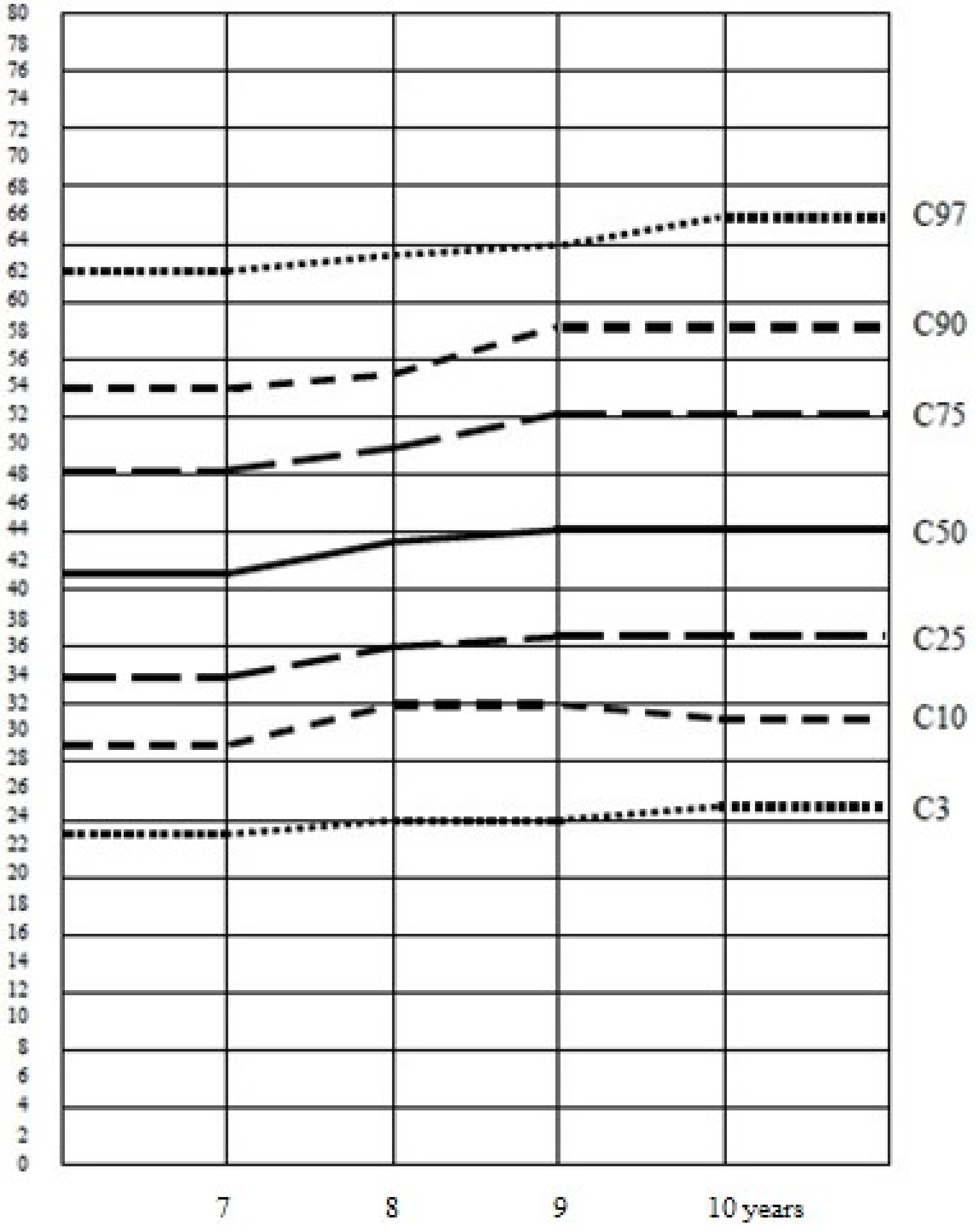

Figure 6. Thoracic Kyphosis Angle for boys (TK)

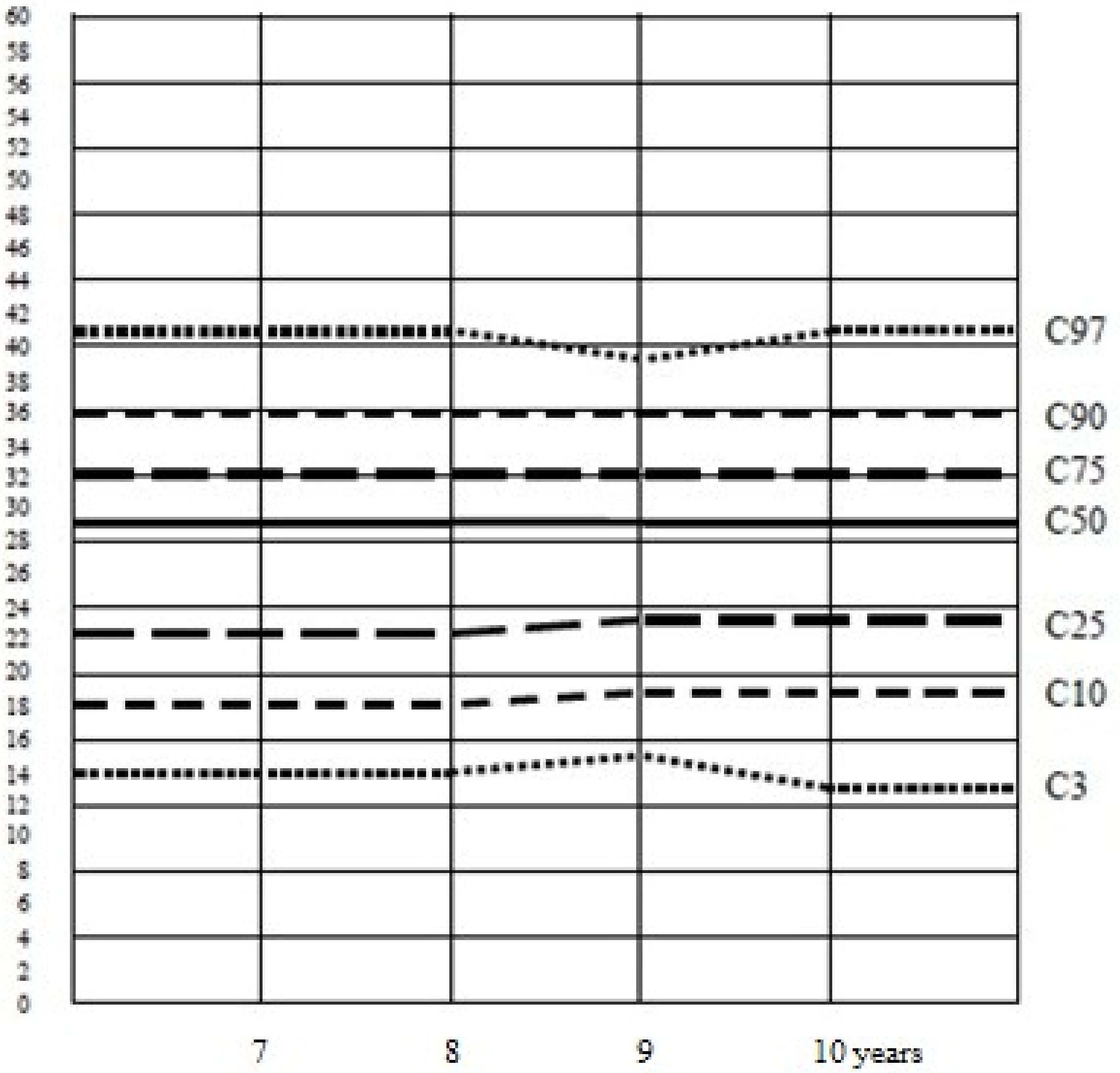

Figure 7. Chest Inclination Angle for girls (CI)

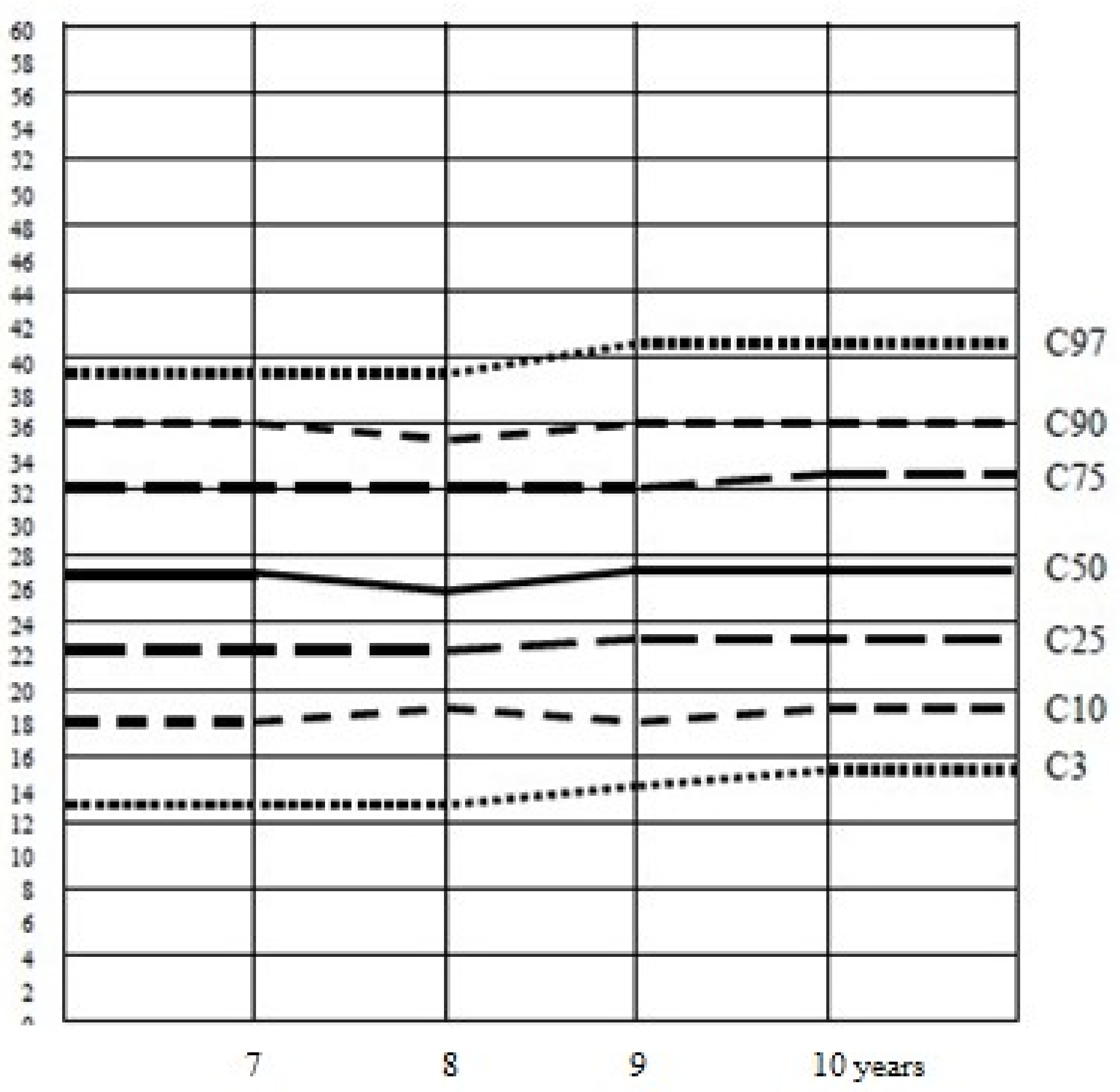

Figure 8. Chest Inclination Angle for boys (CI)

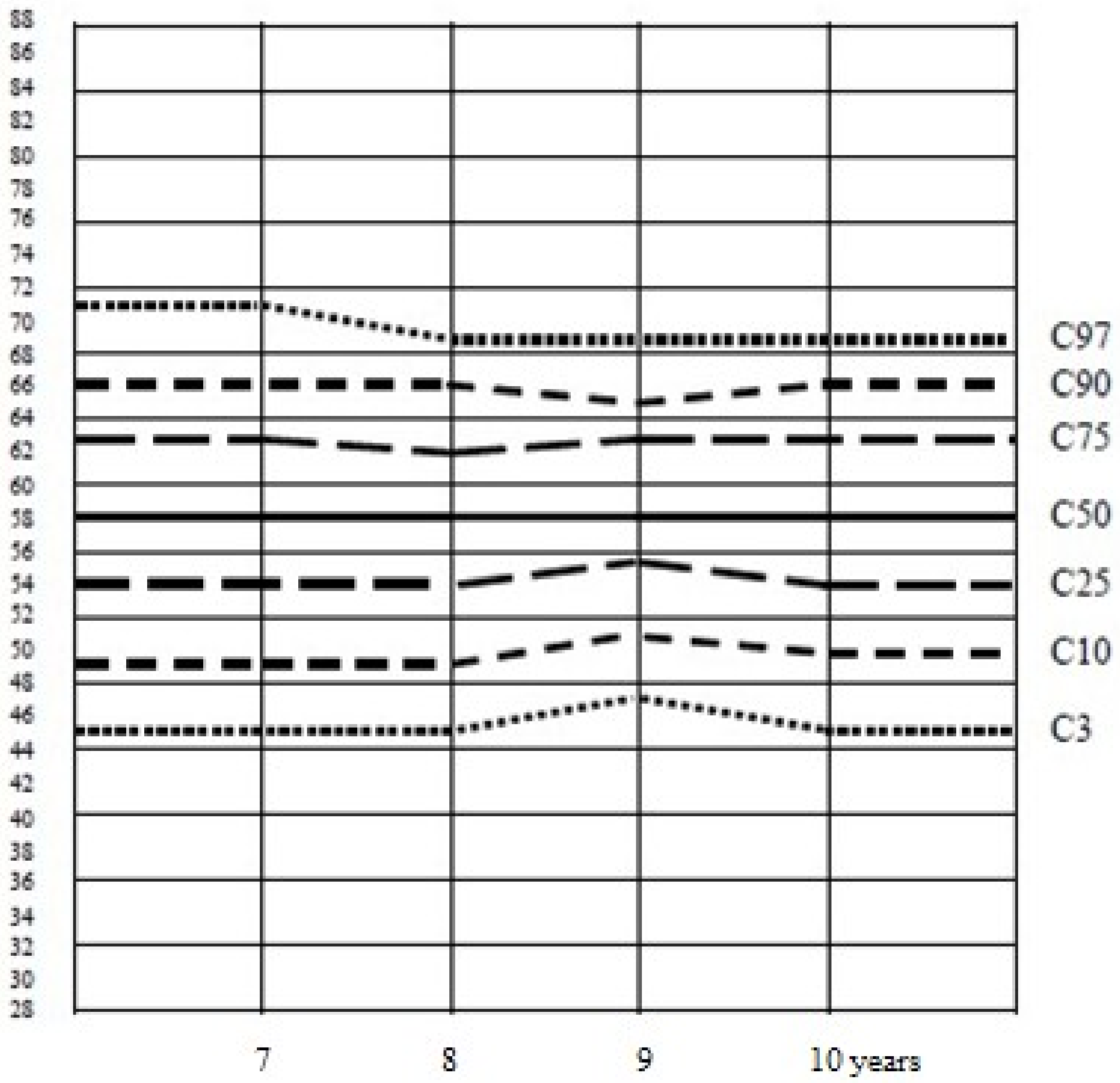

Figure 9. Head Protraction Angle for girls (HP)

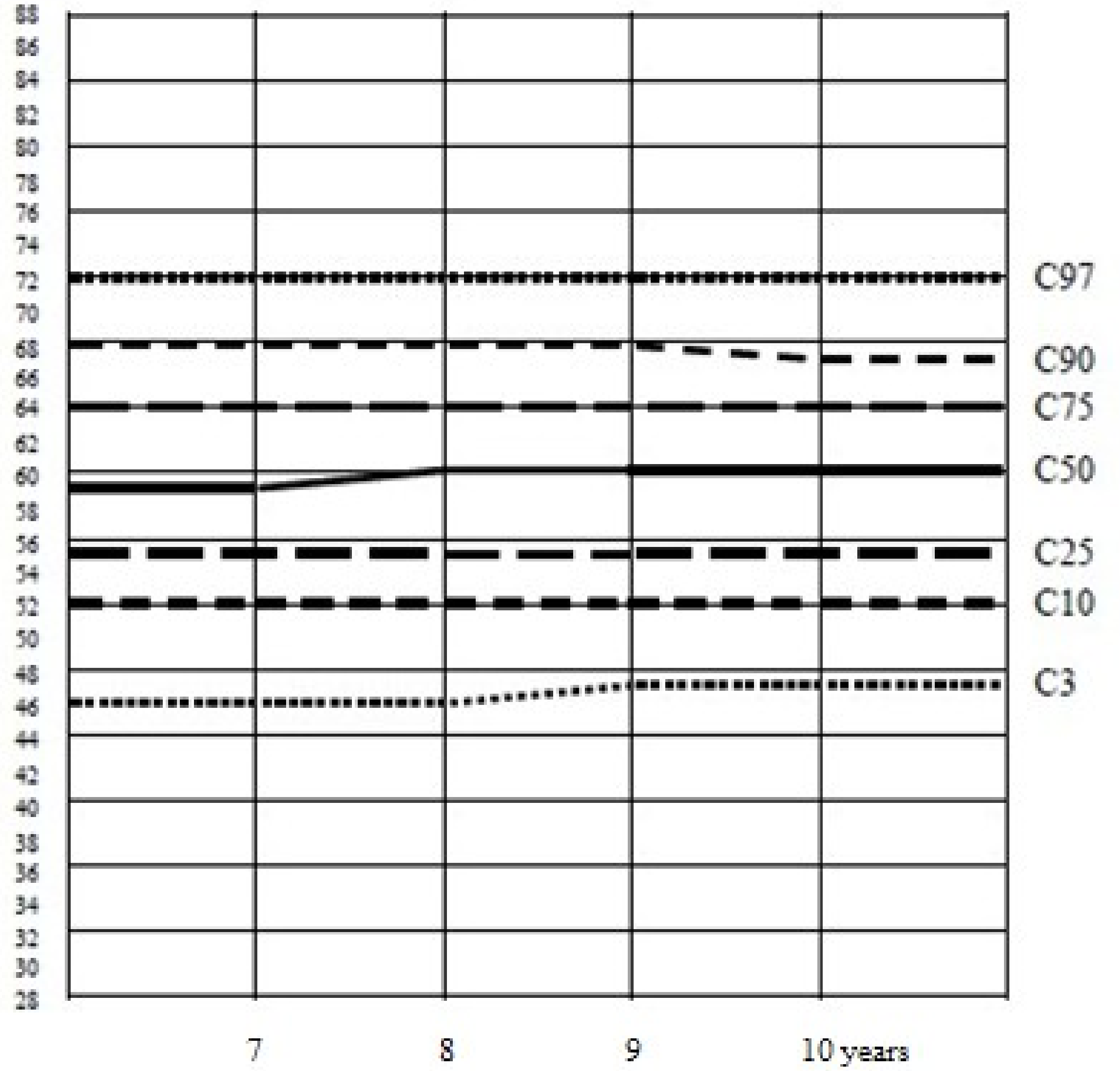

Figure 10. Head Protraction Angle for boys (HP_F)
